# Rapid Quantitation of Adulterants in Premium Marine Oils by Raman and IR Spectroscopy: A Data Fusion Approach

**DOI:** 10.3390/molecules27144534

**Published:** 2022-07-15

**Authors:** Fatema Ahmmed, Daniel P. Killeen, Keith C. Gordon, Sara J. Fraser-Miller

**Affiliations:** 1 Dodd-Walls Centre for Photonic and Quantum Technologies, Department of Chemistry, University of Otago, Dunedin 9016, New Zealand; ahmfa773@student.otago.ac.nz (F.A.); keith.gordon@otago.ac.nz (K.C.G.); 2 Seafood Technologies, The New Zealand Institute for Plant and Food Research Limited, Nelson 7010, New Zealand; daniel.killeen@plantandfood.co.nz

**Keywords:** marine oils, adulteration, Raman spectroscopy, infrared spectroscopy, partial least squares regression

## Abstract

This study uses Raman and IR spectroscopic methods for the detection of adulterants in marine oils. These techniques are used individually and as low-level fused spectroscopic data sets. We used cod liver oil (CLO) and salmon oil (SO) as the valuable marine oils mixed with common adulterants, such as palm oil (PO), omega-3 concentrates in ethyl ester form (O3C), and generic fish oil (FO). We showed that support vector machines (SVM) can classify the adulterant present in both CLO and SO samples. Furthermore, partial least squares regression (PLSR) may be used to quantify the adulterants present. For example, PO and O3C adulterated samples could be detected with a RMSEP value less than 4%. However, the FO adulterant was more difficult to quantify because of its compositional similarity to CLO and SO. In general, data fusion improved the RMSEP for PO and O3C detection. This shows that Raman and IR spectroscopy can be used in concert to provide a useful analytical test for common adulterants in CLO and SO.

## 1. Introduction

Marine oils are a popular dietary supplement, valued for their high concentrations of long chain omega-3, polyunsaturated fatty acids (PUFA), particularly eicosapentaenoic acid (EPA, 20:5), and docosahexaenoic acid (DHA, 22:6) [1]. Refined fish oils (FO) are predominantly produced from rendered oily fish, e.g., anchovy and menhaden; contain >99% triacylglycerols (TAG); and have EPA+DHA concentrations of about 30% total fatty acids [2]. Omega-3 concentrates (O3C) are (usually) produced from marine oils using a combination of transesterification and molecular distillation [3]. This concentrated form can contain more than twice the PUFA of fish oils (FOs) [2], or in the case of the pharmaceuticals Lovaza^®^ and Vascepa^®^, >95% [4]. However, the value of a marine oil is not purely based on its composition, but can stem from factors such as consumer perception, provenance, and historic uses [5]. Cod liver oil (CLO) and salmon oil (SO) benefit from these esoteric, value-adding factors. The relatively high vitamin A content of the former [6] and the astaxanthin content of the latter also contribute to their value [7].

Whenever there is a premium food product, counterfeit versions will often follow [8]. In the case of marine oils, adulteration has been reported for over a century [5]. The detection of adulterated and/or mislabeled marine oils can be extremely difficult due to the similarity between the fatty acid composition of relatively inexpensive FO and premium marine oils. This issue is compounded by the highly variable compositional specifications for marine oils, which are necessary in order to account for seasonal compositional changes in the marine biomass [9]. Sophisticated analytical chemistry methods, such as regiospecific distribution of triacylglycerol fatty acids by nuclear magnetic resonance spectroscopy (NMR) [5], or mass spectrometry (MS)-based lipidomics approaches [10,11] can be used to detect adulteration. Data from these approaches have also been combined with IR spectroscopic data to improve the authentication of cod liver oil [12]. A major advantage of NMR- and MS-based analytical approaches is their selectivity and sensitivity (particularly MS). However, these approaches require expensive instrumentation, a comprehensive library capturing the extent of the compositional variability of the premium oils, and technical expertise in chemistry, i.e., trained operators. 

Vibrational spectroscopic methods have several advantages over NMR- and MS-based analytical techniques. They are rapid, can be performed on solids/liquids with little or no sample preparation, are non-destructive to samples, and are amenable to automation. Furthermore, many instruments are highly portable and, once calibrated, these methods eliminate the need for the use of solvents, making them environmentally friendly [13]. Raman spectroscopy also offers the opportunity to analyze components of interest through packaging. One example is the analysis of FOs through gelatin capsules [2,14], where the successful quantification of pigments, such as carotenoids, were detected at low concentrations [14,15].

IR spectroscopy has been used to identify adulterant oils such as canola, corn, walnut, and soybean oils in cod liver oil [16]. Raman spectroscopy has been used to authenticate FO depending on their omega-3 content (EPA, DHA, and total PUFA) [2,17]. Gao et al. used FT-IR based quantitative analysis (partial least square discriminate analysis (PLS-DA)) to detect terrestrial animal lipid adulterant in FO samples [18]. However, the use of data-fused spectroscopic data and the creation of global models, i.e., a single model that can detect a range of different adulterants in multiple oil types, is relatively unexplored. 

Vibrational spectroscopic techniques combined with multivariate calibration approaches have been used in numerous analytical applications for reliable, in-line, on-line, or at-line analysis. Several research and review manuscripts discuss the potential use of vibrational spectroscopy for the structural analysis of lipids [19,20], quantitative analysis of fatty acids in fish and FO supplements [21,22], authentication and quality parameters of FOs [23], and detection of adulterations in food-based biological samples [24,25]. A tabular representation of the related literature is also shown in the ESI in Table S9.

The present study investigates if IR and Raman spectroscopy can detect and quantitate three different adulterants: palm oil (PO), O3C, and FO in two different premium oils: CLO and SO. We also assessed if (1) quantitation accuracy was improved using the fused data from both vibrational spectroscopic techniques, and (2) whether a single “global” model could be used to accurately quantitate multiple adulterants, in multiple premium oils.

## 2. Materials and Methods

### 2.1. Sample Preparations 

Six different brands of cod liver oil (CLO), six brands of salmon oil (SO), and three different adulterants, including palm oil (PO), omega-3 concentrates in ethyl ester (O3C), and fish oil (FO) were obtained online and from local pharmacies. All of the oil samples were kept at −20 °C to avoid further oxidation or structural changes. For the model sample set (*n* = 108 samples), a series of different weight percentages (0 to 50%) of adulterants (PO, O3C, and FO) were mixed with four batches of cod liver oil (CLO1, CLO3, CLO4, and CLO6) and four batches of salmon oil (SO2, SO3, SO5, and SO6) samples. Two batches of cod liver oil samples (CLO2 and CLO5) and two batches of salmon oil samples (SO1 and SO4) mixed with the adulterants were used to create the independent test set (*n* = 76 samples) to validate the models. The details of the compositional mixtures used in the model and test datasets are given in Table S1.

### 2.2. Raman Spectroscopy

The Raman spectra of the oil samples were measured in triplicate using a MultiRAM Fourier transform (FT) Raman spectrometer (Bruker Optics, Ettlingen, Germany) equipped with a liquid nitrogen-cooled Ge detector (D418T), a Nd:YAG continuous wave laser emitting at 1064 nm, and were controlled using OPUS 7.5 software. The Raman spectra of the oil samples were measured while being contained in glass vials with a 180° backscattering arrangement. The spectra were obtained over the 4000–200 cm^−1^ spectral window with a defocused objective (laser spot size ~2 mm diameter), 300 mW laser power, 4 cm^−1^ resolution, and 128 co-added scans per spectrum. These parameters were selected based on the methods described in earlier works [2,14,26].

### 2.3. ATR-IR Spectroscopy

The Fourier transform infrared (FT-IR) spectra of the samples were obtained using a Vertex 70 spectrometer (Bruker Optics, Ettlingen, Germany) equipped with a GladiATR attenuated total reflectance (ATR) accessory (Pike Technologies, Madison, WI, USA) and were controlled using OPUS 7.5 software. Approximately 10 μL of each sample was placed on the blanked ATR crystal and they were then measured in triplicate. Each spectrum was collected over the spectral window 4000–300 cm^−1^ with 4 cm^−1^ spectral resolution and 32 co-averaged scans. 

### 2.4. Spectral Pre-Processing 

Spectral pre-processing was applied to minimize the sources of variance associated with the sampling arrangements and the non-vibrational spectroscopic light-sample interactions, such as differences in the spectral intensity and baseline effects. 

For the Raman spectra, the spectral windows of 3100–2650 cm^−1^ and 1800–660 cm^−1^ were identified as containing chemically meaningful bands and were selected to undergo pre-processing and subsequent multivariate analysis. Raman data obtained from CLO and SO were baseline corrected using linear baseline correction (LBC) and rubber band correction (RBC), respectively, due to the differing nature of the underlying baseline in the two valuable marine oils. For the combined (CLO and SO) analysis, all of the spectra underwent preprocessing with RBC to ensure consistent preprocessing was applied. Standard normal variate (SNV) transformation was then carried out on the baseline corrected data over the same spectral window for scaling and scatter correction. This combination of pre-processing techniques was selected, as they have been found to be effective across many different materials and systems [27,28,29].

The IR spectra were pre-processed in a similar manner, the spectral windows of 3100–2600 cm^−1^ and 1850–630 cm^−1^ were identified as being chemically meaningful and were preprocessed using LBC followed by SNV [14,26,30]. LBC and SNV was carried out in The Unscrambler X 10.5.1 (CAMO, Oslo, Norway), whereas RBC was carried out in Orange data mining [31].

### 2.5. Data Fusion

The preprocessed Raman and IR spectral data were concatenated into a single matrix prior to undertaking the chemometric analysis. The details of this data fusion method have been described in greater detail elsewhere [14,27,32].

### 2.6. Chemometric Analysis

Qualitative, classification, and quantitative analyses were carried out to evaluate the variation described in the dataset, identify the adulterant present, and quantitate the amount of adulterant present. All of the analyses described here were carried out using the Unscrambler X 10.5.1 (CAMO, Oslo, Norway).

Qualitative analysis was carried out on the individual Raman and IR datasets (all samples) using principal component analysis (PCA), using the preprocessed spectral data as described above (Section 2.4). PCA was calculated with full “leave-one-out” cross-validation and uses the non-linear iterative partial least squares (NIPALS) algorithm.

Support vector machine (SVM) classification was carried out to identify the adulterant present, and to then decide which quantitative model should be applied to an unknown spectrum. The SVM classification models were developed using the model set data with four classes (unadulterated, adulterated with PO, adulterated with O3C, and adulterated with FO). This set of classification models was developed for each individual spectroscopic technique and the fused dataset for both the global (CLO and SO together) and individual (CLO and SO separately) models. The SVM classification was carried out using a C-SVC type SVM with a radial basis function (RBF) (Raman data) or linear type kernel function (IR and fused data) with a gamma coefficient of 1 and a C-value of 100 using the Unscrambler X 10.5.1 (CAMO, Oslo, Norway). 

Partial least square regression (PLSR) models were created for quantitating each adulterant in the oil mixtures. The same spectral range was selected for the PLSR analysis, as described above in Section 2.4. The pre-processed spectral data (X-matrix) were correlated against the adulterant concentration values (% by weight) as the reference data (Y-matrix) using the NIPALS algorithm and systematic (112233) cross-validation with the replicated spectra from each sample removed with each fold. The SVM and PLSR models were evaluated using the independent test set data to obtain classification accuracies (CA) and the root mean squared error of prediction (RMSE_P_) as measures for the model performance.

## 3. Results and Discussion

### 3.1. Raman and Infrared Spectral Features of Oils

**Figure 1:**
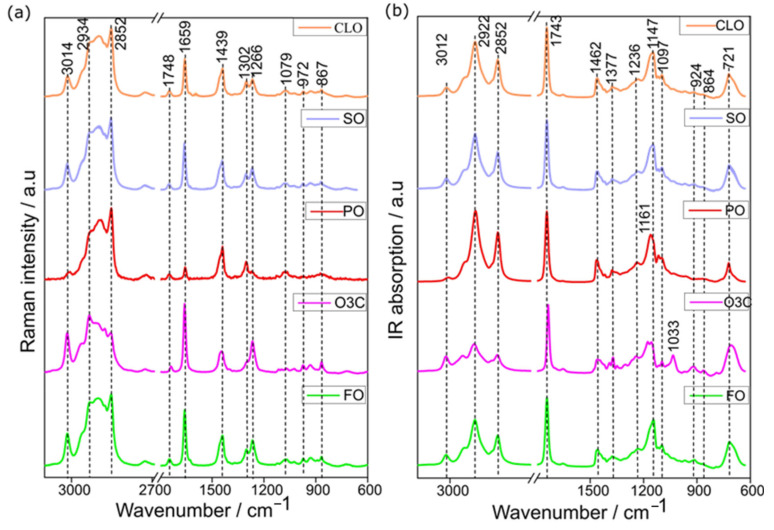
The mean (±Std.Dev) spectra of CLO (*n* = 6 samples), SO (*n* = 6 samples), and the three adulterants studied (palm oil, omega-3 concentrates in ethyl ester, and fish oil) collected using (**a**) Raman and (**b**) IR instruments.

The mean (±standard deviation) Raman (Figure 1a) and IR (Figure 1b) spectra of the premium oil (CLO and SO) and adulterants (PO, O3C, and FO) are presented. The same bands occur in all of the oils, but these vary in both their ratios and intensities, depending on the degree of saturation/unsaturation of their constituent fatty acids [33,34]. The CLO and FO Raman spectra are extremely similar, whereas O3C has a visually greater intensity at 3015 (-C=C-H stretching), 1658 (C=C stretching), and 1268 cm^−1^ (=C-H deformation) [17], reflecting the relative abundance of the omega-3 fatty acids in these oils. Conversely, the Raman spectra of palm oil has an extremely low Raman intensity at these wavenumbers, which is a general feature of (most) terrestrial plant oils [35]. PO had greater relative peak intensities at 2852 cm^−1^ (-CH (CH_2_) stretching), 1439 cm^−1^ (CH scissoring), and 1302 cm^−1^ (-CH_2_, bending). While not intense, all of the Raman spectra contained a carbonyl stretch band, and this was shifted to slightly lower wavenumbers for the O3C, which contained ethyl esters, not triacylglycerols.

The most intense IR bands in both premium oils and adulterants were at 3012 cm^−1^ (-HC=CH- stretching), 2922 cm^−1^ (-CH_2_- stretching), 2852 cm^−1^ (-CH_2_- stretching), and 1743 (C=O stretching). In the same way as that described for the Raman spectra, ethyl esters in the O3C products gave rise to a carbonyl stretching vibration at a slightly lower frequency than the other oils and adulterants. IR vibrational bands at 1236, 1161, and 1097 cm^−1^ (C-O stretching), and 1462 and 1377 cm^−1^ (CH_2_ and CH_3_ bending) were also visible, in good agreement with previous reports of the IR spectra of marine [17,21] and plant derived edible oils [36,37]. The peak intensity at 3012 cm^−1^ was proportional to the unsaturation levels in the oils, with a greater intensity at 2922 and 2852 cm^−1^, indicative of higher levels of saturation [1]. Weak features at 1653 cm^−1^ (C=C stretching), 924 cm^−1^ (=C-H deformation), and 864 cm^−1^ (C-C stretching) were visible in the IR spectra of SO, CLO, O3C, and FO [38,39].

### 3.2. Principal Component Analysis (PCA) 

PCA was carried out on all of the pure samples and their mixtures for the Raman and IR data to explore the inherent variations present in the datasets. 

#### 3.2.1. Raman Analysis

**Figure 2:**
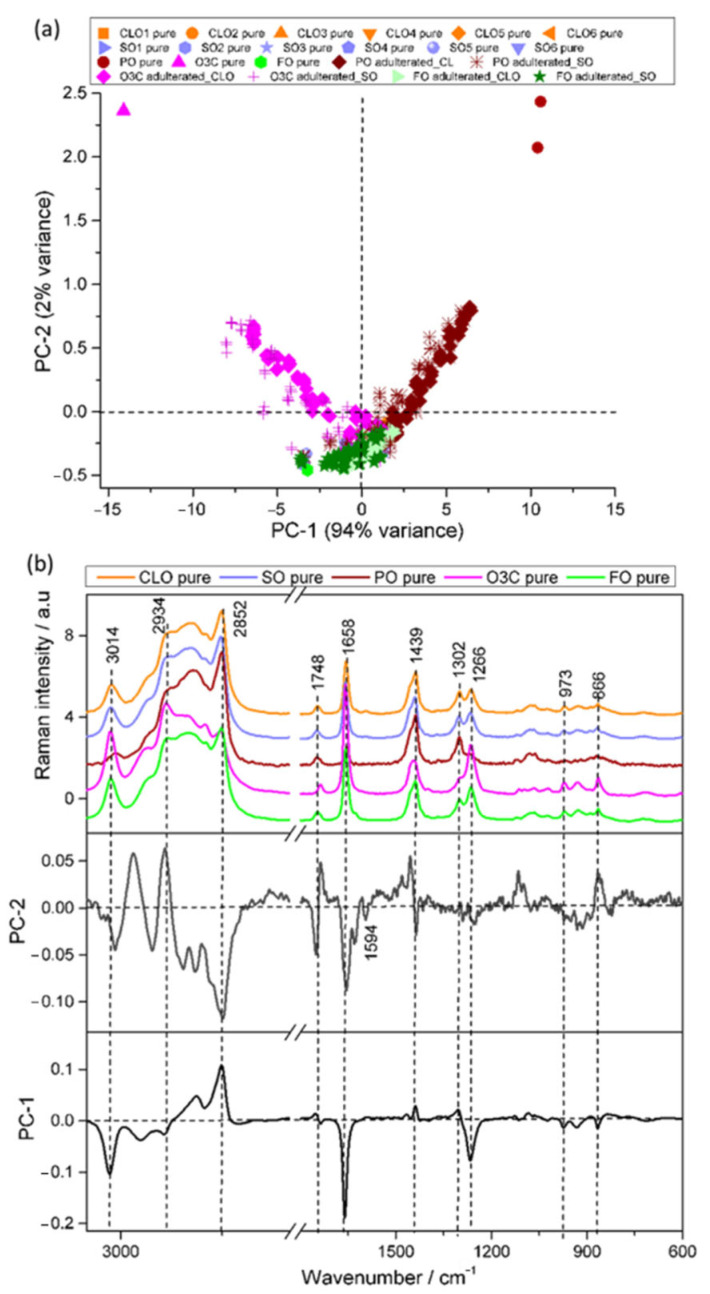
PCA scores and loadings of the Raman spectra collected from the CLO and SO samples adulterated with PO, O3C, and FO. (**a**) PC1 (94% explained variance) versus PC2 (2% explained variance) scores plot, and (**b**) representative Raman spectra and the associated loadings for PCs 1 and 2.

The Raman spectra obtained from the measurement of 6 CLO, 6 SO, 3 adulterants (PO, O3C, and FO), and 144 adulterated mixtures (71 CLO and 71 SO) were subjected to PCA (Figure 2). The first principal component (PC1) described 94% of the total spectral variance across the dataset, with PC2 describing only a minor amount (2%). PC1 separated O3C from PO (Figure 2a). CLO and SO adulterated with PO had increasingly negative PC1 scores, which were proportional to the concentrations of PO adulteration in each sample. The same trend was observed for positive PC1 scores for CLO and SO oils adulterated with increasingly large proportions of O3C. FO adulterated samples tended to cluster with the pure premium marine oils, implying that the spectral variance associated with FO was minimal in this dataset.

The loadings plots presented in Figure 2b highlight the spectral features contributing to the PCA separation. The dominant negative PC1 loadings at 3014, 1658, and 1266 cm^−1^ correspond to unsaturated FA signals, whereas the positive PC1 loading at 2852, 1439, and 1302 cm^−1^ are associated with saturated FAs and other lipid signals. This result suggests that O3C and O3C adulterated samples contained high amounts of omega-3 polyunsaturated fatty acid, whereas PO and PO adulterated samples contained higher amounts of saturated FAs. Negative PC2 loadings were associated with lipid vibrational bands at 2852, 1748, 1658, and 1439 cm^−1^, as well as vitamin A (1594 cm^−1^, all-trans retinol) [40], whereas positive PC2 loadings were attributed to other lipid signals (2934 and 866 cm^−1^). PC2 also described variance due to the shift in the carbonyl stretching vibration between O3C and the other samples, as described in our previous work [2].

#### 3.2.2. IR Analysis 

**Figure 3:**
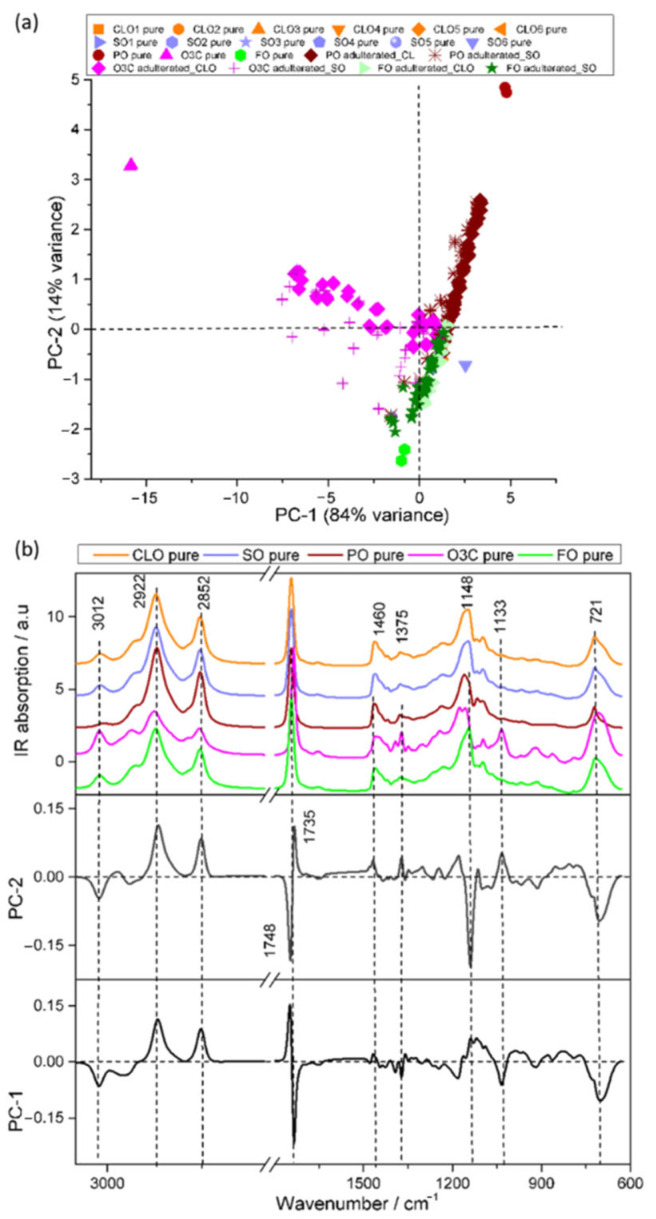
PCA scores and loadings of the IR spectra collected from the CLO and SO samples adulterated with PO, O3C, and FO. (**a**) PC1 (84% explained variance) versus PC2 (14% explained variance) scores plot, and (**b**) representative IR spectra and the associated loadings for PCs 1 and 2.

PCA of IR spectra of the sample set is presented in Figure 3. The first two PCs described 98% of the total spectral variance of the dataset (PC1, 84%; PC2, 14%). The PCA scores plot separated the adulterated samples from the pure samples (Figure 3), in a manner consistent with the PCA of the Raman data. The PO and PO adulterated samples were distributed across a positive PC1 space, with higher PC1 scores indicative of a higher adulterant concentration. The O3C and O3C adulterated samples mirrored this effect in the negative PC1 space. Negative PC2 space contained FO and FO adulterated samples (Figure 3a). This separation was not as strong in the PCA of Raman spectra of the same sample set (Figure 2a). The PO and O3C adulterated samples accounted for more spectral variance than the FO adulterated samples, highlighting the relative similarity of FO to the CLO and SO samples.

Spectral variance responsible for separation of samples in PC-1 and PC-2 are described by the loadings in Figure 3b. The characteristic spectral features in positive PC1 loadings were associated with saturated fatty acids (2922 and 2852 cm^−1^), triacylglycerol carbonyls (1748 cm^−1^), and lipid signatures (1148 cm^−1^). This indicates that the spectral variance in PC1 was mostly derived from PO adulterants. On the other hand, the major spectral features in the negative PC1 loadings could be assigned as unsaturated fatty acids (3012 cm^−1^), ethyl ester carbonyls (1735 cm^−1^), and other lipid signatures (1133, 921, and 704 cm^−1^), corresponding to O3C adulterants. The positive PC2 loadings showed distinctive bands at 2922, 2852, 1735, 1460, 1375, and 1133 cm^−1^, which were associated with the greatest spectral variance, whereas inversely loaded PC2 reflected spectral differences at 3012, 1748, 1148, and 704 cm^−1^ were related to FO, SO, and CLO contributions.

### 3.3. Identification and Quantification of Adulterants

**Figure 4:**
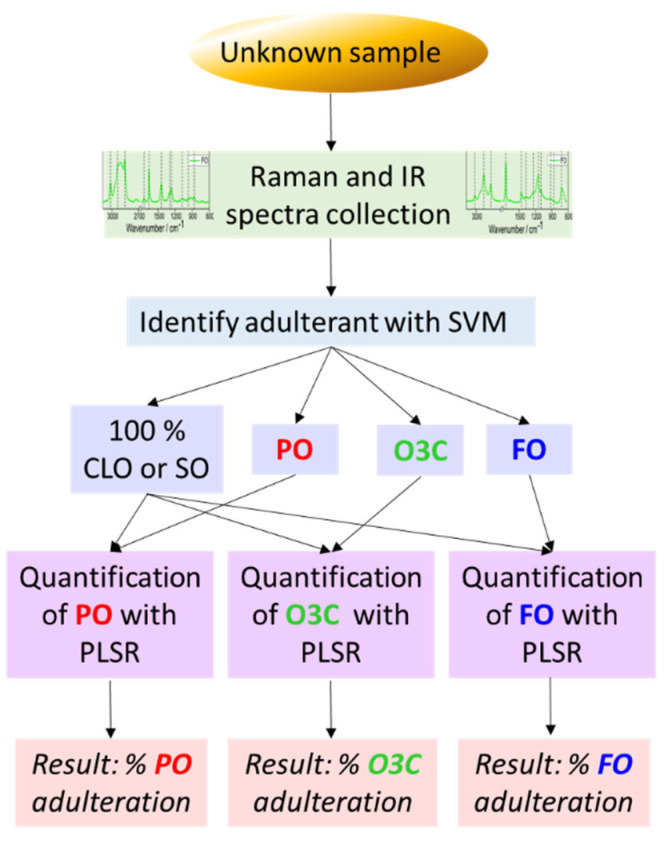
Decision tree/workflow for evaluating unknown samples.

Based on the trends observed in the PCA scores space, it was deemed that this was a promising dataset to explore the identification (classification) and subsequent quantification of adulterants in CLO and SO. First, a classification model was used to categorize the samples based on which the adulterant was present. Next, the categorized data set was modelled to quantitate each adulterant present. The decision tree/workflow for evaluating unknown samples is presented in Figure 4. This workflow was followed for Raman and IR data individually, in addition to the Raman+IR fused dataset. We also assessed if individual models were required for each premium oil type, or if a global model for the detection of adulteration in both CLO and SO was sufficient.

First, the classification methodology (SVM) was applied to the separate spectral data sets (Raman and IR) and also to the fused data set (Raman+IR). All three (Raman, IR, and fused) SVM models for CLO and SO data yielded acceptable calibration (100) and cross validation (Raman: 94%, IR: 99% and low-level fusion: 97%) accuracies for the training dataset. Adulterants in the independent test set was then predicted using the developed SVM models so as to provide insights into the likely performance on unknown samples. 

For Raman, IR, and the Raman and IR fused datasets, the SVM classification model, developed using all of the samples combined, provided a classification accuracy of 76%, 82%, and 85%, respectively, for the independent test dataset (Table 1). The resulting confusion matrix (Table S2), sensitivity, and specificity to individual classes (Table S3) suggested that SVM is an effective method for the classification of the adulterated oil detected in the samples of valuable oil. The SVM model derived from the Raman data (test set accuracy of 76%) was associated with miss-classifications of samples with a mostly low concentration (1–8%, *w*/*w*) of adulterants. The SVM model of the IR data (test set accuracy of 82%) was associated with miss-classifications of samples with low to mid concentrations of mixtures (1–15%, *w*/*w*). The SVM model of the fused data (test set accuracy of 85%) provided the most promising results, with most of the misclassifications in these data being associated with the lowest concentrations (1–8%, *w*/*w*). The SVM classification analysis was also performed for the individual premium oils (CLO and SO) separately (Tables S4–S7). Based on the summary of the confusion matrix, as well as the sensitivity and specificity for the SVM classifications, the model performances were very similar to the global (CLO plus SO) model.

### 3.4. Quantitative Measurements of CLO and SO Adulteration

Partial least square regression (PLSR) analysis was performed to quantitate each adulterant (PO, O3C, and FO) in the CLO and SO sample sets individually, and in a global sample set that contained all CLO and SO samples. PLSR models were developed for each adulterant, allowing for the feasibility of Raman and IR spectroscopy for the quantitation of these adulterants to be assessed both individually (IR and Raman) and as fused spectral data (IR plus Raman). PLSR models were validated using independent test sets: using our prior knowledge of the adulterant type, or pre-screened using the SVM classification model workflow described in Figure 4.

#### 3.4.1. Quantification of PO Adulterant in Cod Liver Oil and Salmon Oil

The performance of each PLSR model developed for quantitating PO in CLO and SO, based on each spectroscopic technique (alone and fused), is given in Table 2. When quantitating the test set data (after SVM screening) using a global model (CLO and SO data combined), the fused data (RMSE_P_ = 3.9%) out-performed the individual techniques (Raman RMSE_P_ = 4.4% and IR RMSE_P_ = 4.6%). When individual valuable oil type models were used instead of a global model, the error could be reduced slightly further. The quantification of PO in CLO was best predicted with a fused model (RMSE_P_ = 2.5%). The quantification of PO in SO was best predicted with IR (RMSE_P_ = 3.4%)

The PLSR calibration models and associated regression coefficients are reported in the ESI for the Raman (Figure S1), IR (Figure S4), and fused models (Figure S7) for completeness. The regression coefficients of all of the models were consistent with separating PO signals (positive coefficients) from valuable oil signals (negative coefficients). The regression coefficients for the global model (Figure S7b) were quite similar, in terms of band position, to the regression coefficient developed from the individual CLO (Figure S7d) and SO models (Figure S7f). 

#### 3.4.2. Spectroscopic Estimation of O3C % in Cod Liver Oil and Salmon Oil

The performance of each PLSR model developed for quantitating O3C in CLO and SO is given in Table 3. When quantitating the test set data (after SVM screening) using a global model (CLO and SO data combined), the fused data (RMSE_P_ = 2.4%) out-performed the individual techniques (Raman RMSE_P_ = 3.4% and IR RMSE_P_ = 3.8%). The individual valuable oil type models preformed slightly better than the global models. The quantification of O3C in CLO was best predicted with either Raman alone or in a fused model, with both giving an RMSE_P_ = 1.5%. The quantification of O3C in SO was best predicted with IR (RMSE_P_ = 1.6%).

The corresponding PLSR and regression coefficients for the quantification of O3C adulterant using Raman, IR, and low-level fusion is presented in Figures S2, S5, and S8, respectively. The positive spectral variance signals at 3016, 2935, 2851, 1267, 1114, and 866 cm^−1^ are associated with lipid signals (Figure S8b), consistent with the regression coefficient obtained from individual Raman data (Figure S2). On the other hand, the negative spectral variance at 1749 and 1660 cm^−1^ could be attributed to ester and unsaturated fatty acids. The positively loaded spectral variance at 3012, 1734, 1371, 1033, and 702 cm^−1^ are also attributed to lipid bands, whereas inversely loaded bands at 1748 and 1140 cm^−1^ could be attributed to the ester bands, which is also in agreement with the individual IR model. The bands at 3016, 1660, 1267, 866, and 1033 cm^−1^ corresponded to the unsaturated fatty acids, which might be mostly the O3C contribution. The global data fusion model (Figure S8b) provided similar spectra in terms of the band position to the individual CLO (Figure S8d) and SO (Figure S8f) model.

#### 3.4.3. Spectroscopic Estimation of FO % in CLO and SO

The detection of FO adulteration in the CLO and SO samples using both global and individual PLSR models was also explored (Table 4). The fused dataset performed best for the global model with RMSE_P_ = 8.6%. When quantitating FO in specific premium marine oils, IR preformed best for detecting FO in CLO (RMSE_P_ = 6.9%) and SO (RMSE_P_ = 6.2%). 

The corresponding PLSR and regression coefficients for the quantification of the FO adulterant using Raman, IR, and low-level fusion, are presented in Figures S3, S6 and S9, respectively. The regression coefficient for the PLSR model with low-level fusion explained 75% of the total variance in the dataset with the first three factors in the global model (Figure S9b), 92% with two factors in CLO, and 78% with two factors in SO. The positively loaded characteristic lipid bands at 3016, 2850, and 1661 cm^−1^ are similar to the regression coefficients of individual Raman spectra (Figure S3), whereas the inversely loaded lipid bands at 3007, 2853, 1460, 1375, 1242, and 1033 cm^−1^ are in agreement with the regression coefficient of the individual IR spectra (Figure S6). The lipid bands (3016, 1661, and 1626 cm^−1^) that corresponded to the unsaturated fatty acids are mostly associated with the FO contribution (Figure S9b). 

The prediction error was higher for the quantification of FO compared with either PO or O3C in the CLO and/or SO samples. This was not unexpected, as variance in the PCA was dominated by PO and O3C signals for both spectroscopic data sets. IR was the only technique that showed some separation with the FO content (Figure 3a), and this was reflected in PLSR model performance (Table 4), with IR being the best performing technique for quantitating FO adulteration. Quantification of FO was not as accurate as quantification of PO and O3C adulteration; however, the approach still be used to gain insight into samples with higher portions of FO adulteration. 

In all instances, the data fused models performed best when quantitating PO, O3C, or FO in the global dataset (RMSE_P_ ranging from 2.4–8.6%).

## 4. Conclusions

This study investigated the feasibility of using Raman and IR spectroscopy to detect and quantitate SO and CLO adulteration with PO, O3C, and FO. Both Raman and IR spectroscopy could detect adulteration with PO and O3C, but could not reach the same levels of accuracy to detect and quantitate adulteration with FO. Neither Raman nor IR spectroscopy were a significantly better technique for this application, but a significant improvement in classification and quantitation could be obtained by combining spectral data from both techniques using low-level data fusion. Furthermore, we present evidence that PO and O3C adulteration can be detected in sample sets containing mixtures of different premium oils, i.e., global models. The described approach may be a less expensive, more rapid alternative to other methods used to detect adulteration in premium marine oils.

## Figures and Tables

**Table 1:** SVM model performance with the accuracy of model set and independent test set using Raman, IR, and the fused dataset.

Technique	Cross Validated Model Set Accuracy (%)	Test Set Accuracy (%)
**Raman**	94	76
**IR**	99	82
**Fused data**	97	85

**Table 2:** Test set performance for PLSR-based quantitative assessment of PO adulteration in CLO and SO.

Instrument Used	Model Name	No. Factors	Prediction (Test Set)				Prediction (Test Set after SVM Classification)			
			r^2^	Slope	Offset	RMSE_P_ (%)	r^2^	Slope	Offset	RMSE_P_ (%)
**Raman (model range: 0 to 50 %)**	CLOSO_PO%	3	0.95	0.84	2.0	4.1	0.95	0.86	1.2	4.4
	CLO_PO%	1	0.96	1	−1.3	3.5	0.98	0.93	1.9	2.6
	SO_PO%	2	0.91	0.76	4.2	5.6	0.88	0.77	4	5.7
**IR (model range: 0 to 50 %)**	CLOSO_PO%	2	0.92	1	−3.5	5.3	0.94	0.99	−2.4	4.6
	CLO_PO%	1	0.94	1	−1.6	4.6	0.96	0.9	3	3.5
	SO_PO%	2	0.97	1	−1.2	3.1	0.96	1	−1.7	3.4
**Low-level fusion (model range: 0 to 50 %)**	CLOSO_PO%	2	0.95	0.91	−0.18	4.3	0.96	0.84	2.1	3.9
	CLO_PO%	1	0.96	1.01	−1.4	3.7	0.98	0.94	1.4	2.5
	SO_PO%	2	0.96	0.92	1.1	3.8	0.95	0.87	3.1	4.1

RMSE_P_ = root means square error of prediction.

**Table 3:** Test set performance for PLSR-based quantitative assessment of O3C adulteration in CLO and SO.

Instrument Used	Model Name	No. Factors	Prediction (Test Set)				Prediction (Test Set after SVM Classification)			
			r^2^	Slope	Offset	RMSE_P_ (%)	r^2^	Slope	Offset	RMSE_P_ (%)
**Raman (model range: 0 to 50 %)**	CLOSO_O3C%	2	0.97	0.91	0.22	3.2	0.97	0.89	0.77	3.4
	CLO_O3C %	1	0.98	0.97	1.2	2.3	0.99	1	−0.8	1.5
	SO_O3C %	2	0.98	0.92	−0.1	2.5	0.97	0.89	0.94	3.4
**IR (model range: 0 to 50 %)**	CLOSO_O3C%	1	0.97	0.92	0.2	3.2	0.96	0.89	1.7	3.8
	CLO_O3C %	1	0.99	0.93	0.02	3.3	0.99	0.95	−0.7	2.1
	SO_O3C %	2	0.99	0.95	−0.4	1.7	0.99	0.95	−0.6	1.6
**Low-level fusion (model range: 0 to 50 %)**	CLOSO_O3C%	2	0.99	0.93	−0.32	2.8	0.99	0.92	0.1	2.4
	CLO_O3C %	1	0.99	0.95	0.54	1.6	0.99	0.98	−0.5	1.5
	SO_O3C %	2	0.99	0.94	−0.3	1.8	0.99	0.93	0.22	1.9

RMSE_P_ = root means square error of prediction.

**Table 4:** Test set performance for the PLSR-based quantitative assessment of FO adulteration in CLO and SO.

Instrument Used	Model Name	No. Factors	Prediction (Test Set)				Prediction (Test Set after SVM Classification)			
			r^2^	Slope	Offset	RMSE_P_ (%)	r^2^	Slope	Offset	RMSE_P_ (%)
**Raman (model range: 0 to 50 %)**	CLOSO_FO%	3	0.75	0.61	3.9	9.3	0.76	0.59	5.3	8.6
	CLO _FO%	1	0.79	0.88	5.9	8.4	0.64	0.83	7.8	10.6
	SO _FO%	3	NA	0.69	−15.6	23	NA	0.64	−11.9	21.8
**IR (model range: 0 to 50 %)**	CLOSO_FO%	2	0.75	0.65	9.3	9.4	0.72	0.66	8.7	9.5
	CLO _FO%	2	0.88	0.62	2.3	6.3	0.85	0.96	−9.9	6.9
	SO _FO%	2	0.88	0.66	5.8	6.5	0.88	0.69	4.9	6.2
**Low-level fusion (model range: 0 to 50 %)**	CLOSO_FO%	3	0.79	0.76	5.2	8.5	0.77	0.75	5.5	8.6
	CLO _FO%	2	0.82	0.89	5.2	7.9	0.77	0.89	5.3	8.5
	SO _FO%	2	0.87	0.71	1.3	6.9	0.79	0.78	−1.4	8.7

RMSE_P_ = root means square error of prediction.

## Data Availability

Data is available on request.

## Supplementary Materials

The following supporting information can be downloaded at: https://www.mdpi.com/article/10.3390/molecules27144534/s1, Table S1: The percentage adulterant composition (by weight) for model and test set samples used in the ternary mixtures of PO, O3C, and FO with CLO and SO; Table S2: SVM model performance for the classification of pure (VO and CLOSO) and adulterants (PO, O3C, and FO) using Raman, IR, and fused data; Table S3: SVM model performance with a sensitivity and specificity test of pure oil samples (CLO and SO) and adulterants (PO, O3C, and FO) using Raman, IR, and fused data; Table S4: SVM model performance for the classification of individual pure oil (CLO) and adulterants (PO, O3C, and FO) using Raman, IR, and fused data; Table S5: SVM model performance with an accuracy, sensitivity, and specificity test of individual pure (CLO) and adulterants (PO, O3C, and FO) using Raman, IR, and fused data; Table S6: SVM model performance for the classification of individual pure oil samples (SO) and adulterants (PO, O3C, and FO) using Raman, IR, and fused data; Table S7: SVM model performance with an accuracy, sensitivity, and specificity test of pure oil samples (SO) and adulterants (PO, O3C, and FO) using Raman, IR, and fused data; Table S8: The model performance accuracy for the PLSR-based quantification of PO, O3C, and FO oils as oil adulterants in CLO and SO; Table S9: Summary table of the related literature and the associated key findings; Figure S1: PLSR calibration lines and regression coefficients for the quantitative prediction of the PO concentration in global (a,b), individual CLO (c,d), and SO (e,f), by Raman; Figure S2: PLSR calibration lines and regression coefficients for the quantitative prediction of the O3C concentration in global (a,b), individual CLO (c,d), and SO (e,f) by Raman; Figure S3: PLSR calibration lines and regression coefficients for the quantitative prediction of the FO concentration in global (a,b), individual CLO (c,d), and SO (e,f) by Raman; Figure S4: PLSR calibration lines and regression coefficients for the quantitative prediction of the PO concentration in global (a,b), individual CLO (c,d), and SO (e,f) by IR; Figure S5: PLSR calibration lines and regression coefficients for the quantitative prediction of the O3C concentration in global (a,b), individual CLO (c,d), and SO (e,f) by IR; Figure S6: PLSR calibration lines and regression coefficients for the quantitative prediction of the FO concentration in global (a,b), individual CLO (c,d), and SO (e,f) by IR; Figure S7: PLSR calibration lines and regression coefficients for the quantitative prediction of the PO concentration in global (a,b), individual CLO, (c,d) and SO (e,f) by low-level fusion; Figure S8: PLSR calibration lines and regression coefficients for the quantitative prediction of the O3C concentration in global (a,b), individual CLO (c,d), and SO (e,f) by low-level fusion; Figure S9: PLSR calibration lines and regression coefficients for the quantitative prediction of the FO concentration in global (a,b), individual CLO (c,d), and SO (e,f) by low-level fusion.

## Sample Availability

Samples are not available as they have been discarded.
