# Supplementary material for: Rapid Quantitation of Adulterants in Premium Marine Oils by Raman and IR Spectroscopy: A Data Fusion Approach
Source: Molecules. 2022 Jul 15;27(14):4534. doi: 10.3390/molecules27144534 (PMC9319805; doi:10.3390/molecules27144534)
Supplement: Supplementary file 1 [file molecules-27-04534-s001.zip › molecules-1818058-supplementary.pdf]

Electronic Supplementary Information for:

Rapid quantitation of adulterants in premium marine oils by Raman and IR spectroscopy: a data fusion approach

Fatema Ahmmed[1], Daniel, P. Killeen[2], Keith C. Gordon[1], Sara J Fraser-Miller[1*]

Contents

Table S1. Summary of the sample mixtures used in this study. Samples are expressed as weight percentages (% w/w). Abbreviations: VO = valuable oil; CLO1 to CLO6 = cod liver oil batch; SO1 to SO6 = salmon oil batch; PO = palm oil; O3C = ω-3 concentrates in ethyl ester; FO = Fish oil; M = model set; T = test set. 2

Table S2. SVM Model performance for classification of pure (VO: CLOSO) and adulterants (PO, O3C and FO) using Raman, IR and Fused data. 4

Table S3. SVM model performance with sensitivity and specificity test of pure oil samples (CLO and SO) and adulterants (PO, O3C and FO) using Raman, IR and Fused data. 5

Table S4. SVM Model performance for classification of individual pure oil (CLO) and adulterants (PO, O3C and FO) using Raman, IR and Fused data. 5

Table S5. SVM model performance with accuracy, sensitivity and specificity test of individual pure (CLO) and adulterants (PO, O3C and FO) using Raman, IR and Fused data. 6

Table S6. SVM Model performance for classification of individual pure oil samples (SO) and adulterants (PO, O3C and FO) using Raman, IR and Fused data. 6

Table S7. SVM model performance with accuracy, sensitivity and specificity test of pure oil samples (SO) and adulterants (PO, O3C and FO) using Raman, IR and Fused data 7

Table S8. The model performance accuracy for PLSR based quantification of PO, O3C and FO oils as oil adulterants in CLO and SO. 8

Table S9 Summary table of related literature and the associated key findings. 9

Figure S1. PLSR calibration lines and regression coefficients for quantitative prediction of PO concentration in global (a,b); individual CLO(c,d) and SO (e,f) by Raman. 13

Figure S2. PLSR calibration lines and regression coefficients for quantitative prediction of O3C concentration in global (a,b); individual CLO (c,d) and SO (e,f) by Raman. 13

Figure S3. PLSR calibration lines and regression coefficients for quantitative prediction of FO concentration in global (a,b); individual CLO(c,d) and SO (e,f) by Raman. 14

Figure S4. PLSR calibration lines and regression coefficients for quantitative prediction of PO concentration in global (a,b); individual CLO (c,d) and SO (e,f) by IR 14

Figure S5. PLSR calibration lines and regression coefficients for quantitative prediction of O3C concentration in global (a,b); individual CLO(c,d) and SO (e,f) by IR. 15

Figure S6. PLSR calibration lines and regression coefficients for quantitative prediction of FO concentration in global (a,b); individual CLO(c,d) and SO (e,f) by IR. 15

Figure S7. PLSR calibration lines and regression coefficients for quantitative prediction of PO concentration in global (a,b); individual CLO (c,d) and SO (e,f) by Low-level fusion. 16

Figure S8. PLSR calibration lines and regression coefficients for quantitative prediction of O3C concentration in global (a,b) ;individual CLO (c,d) and SO (e,f) by Low-level fusion. 16

Figure S9. PLSR calibration lines and regression coefficients for quantitative prediction of FO concentration in global (a,b) ;individual CLO (c,d) and SO (e,f) by Low-level fusion. 17

Table S1. Summary of the sample mixtures used in this study. Samples are expressed as weight percentages (% w/w). Abbreviations: VO = valuable oil; CLO1 to CLO6 = cod liver oil batch; SO1 to SO6 = salmon oil batch; PO = palm oil; O3C = ω-3 concentrates in ethyl ester; FO = Fish oil; M = model set; T = test set.

Predominant oil type	VO (% w/w)	PO (% w/w)	O3C (% w/w)	FO (% w/w)	Batch	Dataset (M or T)
Cod liver oil samples	100.0	0.0	0.0	0.0	CLO3	M
	93.7	0.0	0.0	6.3	CLO3	M
	79.0	0.0	0.0	21.0	CLO3	M
	59.6	0.0	0.0	40.4	CLO3	M
	97.3	0.0	2.7	0.0	CLO3	M
	89.7	0.0	10.3	0.0	CLO3	M
	69.1	0.0	30.9	0.0	CLO3	M
	50.0	0.0	50.0	0.0	CLO3	M
	93.8	6.2	0.0	0.0	CLO3	M
	79.3	20.7	0.0	0.0	CLO3	M
	59.9	40.1	0.0	0.0	CLO3	M
	100.0	0.0	0.0	0.0	CLO4	M
	97.6	0.0	0.0	2.4	CLO4	M
	89.8	0.0	0.0	10.2	CLO4	M
	69.6	0.0	0.0	30.4	CLO4	M
	49.6	0.0	0.0	50.4	CLO4	M
	94.5	0.0	5.5	0.0	CLO4	M
	79.9	0.0	20.1	0.0	CLO4	M
	60.0	0.0	40.0	0.0	CLO4	M
	97.2	2.8	0.0	0.0	CLO4	M
	89.4	10.6	0.0	0.0	CLO4	M
	70.3	29.7	0.0	0.0	CLO4	M
	49.5	50.5	0.0	0.0	CLO4	M
	100.0	0.0	0.0	0.0	CLO6	M
	94.2	0.0	0.0	5.8	CLO6	M
	79.3	0.0	0.0	20.7	CLO6	M
	60.0	0.0	0.0	40.0	CLO6	M
	99.1	0.0	0.9	0.0	CLO6	M
	89.6	0.0	10.4	0.0	CLO6	M
	69.8	0.0	30.2	0.0	CLO6	M
	50.6	0.0	49.4	0.0	CLO6	M
	93.7	6.3	0.0	0.0	CLO6	M
	79.8	20.2	0.0	0.0	CLO6	M
	59.5	40.5	0.0	0.0	CLO6	M
	100.0	0.0	0.0	0.0	CLO1	M
	98.5	0.0	0.0	1.5	CLO1	M
	89.6	0.0	0.0	10.4	CLO1	M
	69.7	0.0	0.0	30.3	CLO1	M
	51.2	0.0	0.0	48.8	CLO1	M
	94.7	0.0	5.3	0.0	CLO1	M
	89.0	0.0	11.0	0.0	CLO1	M
	60.6	0.0	39.4	0.0	CLO1	M
	98.3	1.7	0.0	0.0	CLO1	M
	89.2	10.8	0.0	0.0	CLO1	M
	69.9	30.1	0.0	0.0	CLO1	M
	49.9	50.1	0.0	0.0	CLO1	M
	100.0	0.0	0.0	0.0	CLO2	T
	98.4	0.0	0.0	1.6	CLO2	T
	89.6	0.0	0.0	10.4	CLO2	T
	74.6	0.0	0.0	25.4	CLO2	T
	57.6	0.0	0.0	42.4	CLO2	T
	50.4	0.0	0.0	49.6	CLO2	T
	98.1	0.0	1.9	0.0	CLO2	T
	91.8	0.0	8.2	0.0	CLO2	T
	74.9	0.0	25.1	0.0	CLO2	T
	65.4	0.0	34.6	0.0	CLO2	T
	49.7	0.0	50.3	0.0	CLO2	T
	97.6	2.4	0.0	0.0	CLO2	T

Predominant oil type	VO (% w/w)	PO (% w/w)	O3C (% w/w)	FO (% w/w)	Batch	Dataset (M or T)
Salmon oil	100.0	0.0	0.0	0.0	SO2	M
	95.1	0.0	0.0	4.9	SO2	M
	80.1	0.0	0.0	19.9	SO2	M
	60.1	0.0	0.0	39.9	SO2	M
	94.3	0.0	5.7	0.0	SO2	M
	80.0	0.0	20.0	0.0	SO2	M
	60.0	0.0	40.0	0.0	SO2	M
	99.0	1.0	0.0	0.0	SO2	M
	90.1	9.9	0.0	0.0	SO2	M
	70.3	29.7	0.0	0.0	SO2	M
	50.0	50.0	0.0	0.0	SO2	M
	100.0	0.0	0.0	0.0	SO3	M
	98.5	0.0	0.0	1.5	SO3	M
	90.1	0.0	0.0	9.9	SO3	M
	70.1	0.0	0.0	29.9	SO3	M
	49.8	0.0	0.0	50.2	SO3	M
	98.9	0.0	1.1	0.0	SO3	M
	89.8	0.0	10.2	0.0	SO3	M
	70.3	0.0	29.7	0.0	SO3	M
	50.3	0.0	49.7	0.0	SO3	M
	95.0	5.0	0.0	0.0	SO3	M
	79.8	20.2	0.0	0.0	SO3	M
	60.0	40.0	0.0	0.0	SO3	M
	100.0	0.0	0.0	0.0	SO5	M
	95.2	0.0	0.0	4.8	SO5	M
	80.2	0.0	0.0	19.8	SO5	M
	59.9	0.0	0.0	40.1	SO5	M
	95.0	0.0	5.0	0.0	SO5	M
	80.0	0.0	20.0	0.0	SO5	M
	60.0	0.0	40.0	0.0	SO5	M
	98.9	1.1	0.0	0.0	SO5	M
	89.5	10.5	0.0	0.0	SO5	M
	70.3	29.7	0.0	0.0	SO5	M
	50.0	50.0	0.0	0.0	SO5	M
	100.0	0.0	0.0	0.0	SO6	M
	98.8	0.0	0.0	1.2	SO6	M
	89.8	0.0	0.0	10.2	SO6	M
	69.7	0.0	0.0	30.3	SO6	M
	50.3	0.0	0.0	49.7	SO6	M
	99.1	0.0	0.9	0.0	SO6	M
	89.8	0.0	10.2	0.0	SO6	M
	69.9	0.0	30.1	0.0	SO6	M
	50.0	0.0	50.0	0.0	SO6	M
	94.1	5.9	0.0	0.0	SO6	M
	80.2	19.8	0.0	0.0	SO6	M
	60.1	39.9	0.0	0.0	SO6	M
	100.0	0.0	0.0	0.0	SO1	T
	98.8	0.0	0.0	1.2	SO1	T
	92.6	0.0	0.0	7.4	SO1	T
	74.6	0.0	0.0	25.4	SO1	T
	64.6	0.0	0.0	35.4	SO1	T
	49.9	0.0	0.0	50.1	SO1	T
	98.8	0.0	1.2	0.0	SO1	T
	84.7	0.0	15.3	0.0	SO1	T
	57.1	0.0	42.9	0.0	SO1	T
	49.2	0.0	50.8	0.0	SO1	T
	98.2	1.8	0.0	0.0	SO1	T
	85.3	14.7	0.0	0.0	SO1	T

	88.9	11.1	0.0	0.0	CLO2	T
	74.7	25.3	0.0	0.0	CLO2	T
	57.1	42.9	0.0	0.0	CLO2	T
	50.9	49.2	0.0	0.0	CLO2	T
	100.0	0.0	0.0	0.0	CLO5	T
	97.6	0.0	0.0	2.4	CLO5	T
	91.8	0.0	0.0	8.2	CLO5	T
	74.2	0.0	0.0	25.8	CLO5	T
	64.5	0.0	0.0	35.5	CLO5	T
	52.0	0.0	0.0	48.0	CLO5	T
	98.6	0.0	1.4	0.0	CLO5	T
	89.3	0.0	10.7	0.0	CLO5	T
	74.6	0.0	25.4	0.0	CLO5	T
	57.2	0.0	42.8	0.0	CLO5	T
	49.5	0.0	50.5	0.0	CLO5	T
	97.9	2.1	0.0	0.0	CLO5	T
	91.9	8.1	0.0	0.0	CLO5	T
	74.9	25.1	0.0	0.0	CLO5	T
	64.1	35.9	0.0	0.0	CLO5	T
	50.0	50.0	0.0	0.0	CLO5	T
	73.3	26.7	0.0	0.0	SO1	T
	57.3	42.7	0.0	0.0	SO1	T
	50.2	49.8	0.0	0.0	SO1	T
	100.0	0.0	0.0	0.0	SO4	T
	98.8	0.0	0.0	1.2	SO4	T
	85.3	0.0	0.0	14.7	SO4	T
	75.2	0.0	0.0	24.8	SO4	T
	57.5	0.0	0.0	42.5	SO4	T
	50.1	0.0	0.0	49.9	SO4	T
	98.8	0.0	1.2	0.0	SO4	T
	92.7	0.0	7.3	0.0	SO4	T
	75.1	0.0	24.9	0.0	SO4	T
	65.0	0.0	35.0	0.0	SO4	T
	50.3	0.0	49.7	0.0	SO4	T
	98.3	1.7	0.0	0.0	SO4	T
	91.6	8.4	0.0	0.0	SO4	T
	75.1	24.9	0.0	0.0	SO4	T
	64.7	35.3	0.0	0.0	SO4	T
	49.9	50.1	0.0	0.0	SO4	T
Pure adulterants	0.0	0.0	0.0	100.0	FO	
	0.0	0.0	100.0	0.0	O3C	
	0.0	100.0	0.0	0.0	PO	

Table S2. SVM Model performance for classification of pure (VO: CLOSO) and adulterants (PO, O3C and FO) using Raman, IR and Fused data.

Raman Test set (76% accuracy)					
Confusion matrix	Actual	1	2	3	4
Predicted		CLOSO	PO	O3C	FO
1	CLOSO	21	1	0	0
2	PO	0	45	3	11
3	O3C	6	6	45	2
4	FO	9	3	9	47
**IR Test set (82% accuracy)**					
Confusion matrix	Actual	1	2	3	4
Predicted		CLSO	PO	O3C	FO
1	CLOSO	27	3	4	6
2	PO	0	47	3	0
3	O3C	0	0	47	0
4	FO	9	10	3	54

Fused data Test set (85% accuracy)					
Confusion matrix	Actual	1	2	3	4
Predicted		CLOSO	PO	O3C	FO
1	CLOSO	24	3	4	0
2	PO	0	49	3	1
3	O3C	0	0	46	0
4	FO	12	5	4	59

Table S3. SVM model performance with sensitivity and specificity test of pure oil samples (CLO and SO) and adulterants (PO, O3C and FO) using Raman, IR and Fused data.

Technique	Test set							
	Sensitivity to individual classes (%)				Specificity to individual classes (%)			
	CLOSO	PO	O3C	FO	CLOSO	PO	O3C	FO
Raman	58	82	79	78	99	91	91	86
IR	75	78	82	90	93	98	100	86
Fused data	67	86	82	98	96	97	100	86

Table S4. SVM Model performance for classification of individual pure oil (CLO) and adulterants (PO, O3C and FO) using Raman, IR and Fused data.

Raman Test set (81% accuracy)					
Confusion matrix	Actual	1	2	3	4
Predicted		CLO	PO	O3C	FO
1	CLO	9	0	0	0
2	PO	0	26	0	0
3	O3C	0	0	23	0
4	FO	9	4	7	37
**IR Test set (78% accuracy)**					
Confusion matrix	Actual	1	2	3	4
Predicted		CLO	PO	O3C	FO
1	CLO	5	0	1	0
2	PO	0	23	0	0
3	O3C	0	0	26	0
4	FO	13	7	3	30
**Fused data Test set (74% accuracy)**					
Confusion matrix	Actual	1	2	3	4
Predicted		CLO	PO	O3C	FO
1	CLO	3	0	0	0
2	PO	0	23	0	0
3	O3C	0	0	24	0
4	FO	15	7	6	30

Table S5. SVM model performance with accuracy, sensitivity and specificity test of individual pure (CLO) and adulterants (PO, O3C and FO) using Raman, IR and Fused data.

Technique	Training set		Test set								
	Accuracy (calibration)	Accuracy (cross validation)	Accuracy	Sensitivity to individual classes (%)				Specificity to individual classes (%)			
				CLO	PO	O3C	FO	CLO	PO	O3C	FO
**Raman**	100	95	81	50	87	77	100	100	100	100	74
**IR**	100	99	78	28	77	87	100	99	100	100	71
**Fused data**	100	96	74	17	77	80	100	100	92	100	64

Table S6. SVM Model performance for classification of individual pure oil samples (SO) and adulterants (PO, O3C and FO) using Raman, IR and Fused data.

**Raman Test set (66% accuracy)**					
Confusion matrix	Actual	1	2	3	4
Predicted		SO	PO	O3C	FO
1	SO	18	5	1	0
2	PO	0	24	9	20
3	O3C	0	1	17	0
4	FO	0	0	0	10
**IR Test set (73% accuracy)**					
Confusion matrix	Actual	1	2	3	4
Predicted		SO	PO	O3C	FO
1	SO	18	0	0	0
2	PO	4	26	0	0
3	O3C	3	3	21	0
4	FO	18	0	0	12
**Fused data Test set (71% accuracy)**					
Confusion matrix	Actual	1	2	3	4
Predicted		SO	PO	O3C	FO
1	SO	15	5	0	1
2	PO	0	19	7	8
3	O3C	0	5	20	0
4	FO	3	1	0	21

Table S7. SVM model performance with accuracy, sensitivity and specificity test of pure oil samples (SO) and adulterants (PO, O3C and FO) using Raman, IR and Fused data

**Technique**	**Training set**		**Test set**									
	Accuracy (calibration)	Accuracy (cross validation)	Accuracy	Sensitivity to individual classes (%)				Specificity to individual classes (%)				
				SO	PO	O3C	FO	SO	PO	O3C	FO
**Raman**	100	95	66	100	80	63	33	93	61	99	100
**IR**	100	99	73	100	87	77	40	71	96	100	100
**Fused data**	100	97	71	83	63	77	70	93	80	194	95

Table S8. The model performance accuracy for PLSR based quantification of PO, O3C and FO oils as oil adulterants in CLO and SO.

Instrument used		No. factors	Calibration		Cross-validation		Prediction (test set)				Prediction (test set) SVM			
			r^2	$RMSE_C$ (%)	r^2	$RMSE_{cv}$ (%)	r^2	slope	offset	$RMSE_P$ (%)	r^2	slope	offset	$RMSE_P$ (%)
**PO adulterated samples**														
**Raman (model range: 0 to 50 %)**	CLOSO_PO%	3	0.95	4.8	0.94	5.9	*0.95*	*0.84*	*2.0*	*4.1*	*0.95*	*0.86*	*1.2*	*4.4*
	CLO_PO%	1	0.98	3.4	0.97	3.9	*0.96*	*1.0*	*-1.3*	*3.5*	*0.98*	*0.93*	*1.9*	*2.6*
	SO_PO%	2	0.93	6.4	0.92	6.7	*0.91*	*0.76*	*4.2*	*5.6*	*0.88*	*0.77*	*4.0*	*5.7*
**IR (model range: 0 to 50 %)**	CLOSO_PO%	2	0.98	2.7	0.97	4.1	*0.92*	*1.0*	*-3.5*	*5.3*	*0.94*	*0.99*	*-2.4*	*4.6*
	CLO_PO%	1	0.97	3.8	0.97	4.5	*0.94*	*1.0*	*-1.6*	*4.6*	*0.96*	*0.90*	*3.0*	*3.5*
	SO_PO%	2	0.99	1.7	0.99	2.4	*0.97*	*1.0*	*-1.2*	*3.1*	*0.96*	*1.0*	*-1.7*	*3.4*
**Low-level fusion (model range: 0 to 50 %)**	CLOSO_PO%	2	0.96	4.5	0.96	4.7	*0.95*	*0.91*	*-0.18*	*4.3*	*0.96*	*0.84*	*2.1*	*3.9*
	CLO_PO%	1	0.98	3.4	0.97	3.9	*0.96*	*1.01*	*-1.4*	*3.7*	*0.98*	*0.94*	*1.4*	*2.5*
	SO_PO%	2	0.99	2.7	0.99	2.9	*0.96*	*0.92*	*1.1*	*3.8*	*0.95*	*0.87*	*3.1*	*4.1*
**O3C adulterated samples**														
**Raman (model range: 0 to 50 %)**	CLOSO_O3C%	2	0.96	4.5	0.96	5.0	*0.97*	*0.91*	*0.22*	*3.2*	*0.97*	*0.89*	*0.77*	*3.4*
	CLO _ O3C %	1	0.99	2.1	0.99	2.2	*0.98*	*0.97*	*1.2*	*2.3*	*0.99*	*1.0*	*-0.8*	*1.5*
	SO _ O3C %	2	0.98	3.0	0.98	3.5	*0.98*	*0.92*	*-0.1*	*2.5*	*0.97*	*0.89*	*0.94*	*3.4*
**IR (model range: 0 to 50 %)**	CLOSO_O3C%	1	0.95	4.4	0.95	4.5	*0.97*	*0.92*	*0.2*	*3.2*	*0.96*	*0.89*	*1.7*	*3.8*
	CLO _ O3C %	1	0.99	1.6	0.99	1.9	*0.99*	*0.93*	*0.02*	*3.3*	*0.99*	*0.95*	*-0.7*	*2.1*
	SO _ O3C %	2	0.99	1.9	0.99	2.4	*0.99*	*0.95*	*-0.4*	*1.7*	*0.99*	*0.95*	*-0.6*	*1.6*
**Low-level fusion (model range: 0 to 50 %)**	CLOSO_O3C%	2	0.99	2.0	0.99	2.3	*0.99*	*0.93*	*-0.32*	*2.8*	*0.99*	*0.92*	*0.10*	*2.4*
	CLO _ O3C %	1	0.99	1.6	0.99	1.7	*0.99*	*0.95*	*0.54*	*1.6*	*0.99*	*0.98*	*-0.50*	*1.5*
	SO _ O3C %	2	0.99	2.0	0.99	2.5	*0.99*	*0.94*	*-0.3*	*1.8*	*0.99*	*0.93*	*0.22*	*1.9*
**FO adulterated samples**														
**Raman (model range: 0 to 50 %)**	CLOSO_FO%	3	0.72	12.5	0.59	15.4	*0.75*	*0.61*	*3.9*	*9.3*	*0.76*	*0.59*	*5.3*	*8.6*
	CLO _FO%	1	0.92	6.6	0.91	7.7	*0.79*	*0.88*	*5.9*	*8.4*	*0.64*	*0.83*	*7.8*	*10.6*
	SO _FO%	3	0.85	9.1	0.45	18.0	*NA*	*0.69*	*-15.6*	*23*	*NA*	*0.64*	*-11.9*	*21.8*
**IR (model range: 0 to 50 %)**	CLOSO_FO%	2	0.67	13.4	0.62	14.8	*0.75*	*0.65*	*9.3*	*9.4*	*0.72*	*0.66*	*8.7*	*9.5*
	CLO _FO%	2	0.94	5.5	0.93	6.5	*0.88*	*0.62*	*2.3*	*6.3*	*0.85*	*0.96*	*-9.9*	*6.9*
	SO _FO%	2	0.74	12.0	0.65	14.3	*0.88*	*0.66*	*5.8*	*6.5*	*0.88*	*0.69*	*4.9*	*6.2*
**Low level fusion (model range: 0 to 50 %)**	CLOSO_FO%	3	0.81	10.1	0.75	12.0	*0.79*	*0.76*	*5.2*	*8.5*	*0.77*	*0.75*	*5.5*	*8.6*
	CLO _FO%	2	0.94	5.9	0.92	6.7	*0.82*	*0.89*	*5.2*	*7.9*	*0.77*	*0.89*	*5.3*	*8.5*
	SO _FO%	2	0.78	11.0	0.58	15.7	*0.87*	*0.71*	*1.3*	*6.9*	*0.79*	*0.78*	*-1.4*	*8.7*

$RMSE_C$ = Root means square error of calibration, RMSEcv = Root means square error of cross-validation and RMSEp = Root means square error of prediction.

Table S9 Summary table of related literature and the associated key findings.

**Application scope**	**Samples studied**	**Technique**	**Objective**	**Main findings**	**References**
Biochemical composition	King salmon, Greenshell muscle	FT-NIR	Rapid analysis of composition (protein, fat, moisture, carbohydrate and ash)	- FT-NIR model performed well to quantify the moisture, protein and fat content where the coefficient of determination (r^{2}) and RMSECV value are found more than 0.96 and less than 1.09%, respectively<br>- FT-NIR analysis reported as a suitable method for research and industrial application	[1]
	Pacific oyster (*Crassostrea gigas*), Rock oyster (*Saccostrea glomerata*)	near-infrared IR reflectance spectroscopy (NIRS)	Estimation of composition (protein, moisture, fat and glycogen) in oyster	- NIRS model quantified proximate composition for both Pacific oyster and Rock oyster with high prediction accuracy (r^2 = 0.96, 0.97) and low RPD (4.8, 5.5), respectively	[2]
	Freshwater mollusk	Confocal Raman microscope (CRS)	Identification of mollusks shell composition at microstructure level and determination of relationship between composition and growth	- CRS successfully measured the shell growth and confirmed the polymorph of calcium carbonate, aragonite and vaterite distribution in each mollusc shell	[3]
	Rainbow Trout	Raman microscope	-Determination of fatty acid composition in visceral adipose tissue	- Predicted high value for ALA (α -linolenic acid), LA (linoleic acid), EPA and DHA<br>- Developed calibration model to predict PUFAs contents for large scale and high throughput phenotype	[4]
	Fillet from farmed and wild salmon	FT-Raman	- Identification of carotenoid, collagen and fat in fish muscle<br>- Relative concentration analysis of the compound	- Contained detailed spectral information of the target compounds and relative concentration	[5]
	Salmon oil	Near-infrared (NIR)	- Determination of fatty acid and lipid classes	- PLS-NIR method quantified several fatty acids, including PUFA, MUFA, omega-3, omega-6, palmitic acid, oleic acid, alpha-linolenic acid<br>- Quantified several lipids classed (free fatty acid, diacylglycerides and triacylglycerides)<br>- RRMSEP ≤1.41%	[6]
	Fish oil	FT-Raman	- Quantification of PUFA,<br>- Detection of ethyl ester and lipid oxidation	- Measured successfully the PUFA content directly through capsules<br>- Detected fat oxidation with high sensitivity<br>- Separated oil depending on the PV value	[7]

	Salmon (*Salmo salar*)	Raman spectroscopy	- Investigation of iodine values,<br>- Characterization of FAs from different sample regimes (ground samples, intact salmon muscle and oil extracts)	- Provided better iodine value predictions<br>- Raman could be a potential method of rapid analysis of fatty acid unsaturation of salmon.	[8]
	Fish oil capsules	FT-IR, NIR and Raman spectroscopy	- Investigation of the feasibility of FT-IR, NIR and Raman spectroscopy for quantitative analysis of total omega-3 including EPA and DHA	- FT-IR, NIR and Raman spectroscopy combined with PLSR, identified omega-3 including EPA and DHA<br>- NIR spectra provided models with good performances and predicted EPA (r^2 = 0.979, SECV =2.43%, and SEP =3.11%) and DHA (r^2 = 0.972, SECV = 2.34%, and SEP = 2.60%)	[9]
Classification / detection of adulterant/ authentication	Patin fish oil (PFO)	FT-IR	- Detection of PFO adulterated with palm oil (PO)	- PLS and PCR model quantified PO adulterant in PFO. RMSEC: 0.77–5.50% RMSEP: 2.07–3.59% R^2: 0.99<br>- Separated pure PFO and PFO adulterated with PO correctly (100%, accuracy)	[10]
	Fish oil capsules	ATR-FTIR and FT-Raman	- Classification of lipids class as ethyl ester (EE), triglyceride (TAG)	- TAG containing fish oil were clearly separated (100%) from EE containing fish oil<br>-PLS-DA model for FT-IR data predicted better prediction (r^2 = 0.99, lower errors RMSEP = 0.02%) of EE and TAG compared to FT-Raman (r^2 = 0.95, RMSEP = 0.11%)	[11]
	3 Tuna species: *Thunnus albacares*, *T. obesus Katsuwonus pelamis*	Mid-infrared spectroscopy (MIR)	-Identification of 3 different species of tuna (bigeye, yellowfin and skipjack)<br>-discrimination of various binary mixture of canned tunas	- calibration model accounted 100% accuracy for skipjack tuna, while misclassification was recorded for bigeye and yellowfin cans<br>- MIR data showed 20 out of 30 commercial tuna cans were mislabelled. The model accounted 90.38% accuracy for validation set	[12]
	Black carp (n=100), Grass carp (n=100), Silver carp (n=100), Bighead carp (n=100), Common carp (n=80), Crucian (n=100) , and Bream (n=70)	NIR	- Differentiation of freshwater fish species (Cyprinids)<br>- Evaluation of linear discriminate analysis LDA classification performance	- Principal component analysis (PCA-LDA) analysis based on MSC preprocessing evaluated the highest (100%) accuracy, specificity, sensitivity, and precision value in the parameters<br>- PCA–LDA and Fast Fourier transformation (FFT–LDA) model showed 100% prediction accuracy for sample classifications.	[13]
	90 canned fish in variety of oil	FT-IR	- Identification of packing oil authenticity from commercial canned tuna and other fish	- PCA analysis clearly classified olive oil from other seed oils except high-oleic sunflower oil (HOSO)<br>- PLS-DA accurately differentiated HOSO from other oils with 100% sensitivity and specificity	[14]

				- PLS-DA also separated EVOO from OO with acceptable prediction value (sensitivity= 93%, specificity= 97%)	
	Red mullet<br>Atlantic mullet<br>Plaice<br>Flounder<br>Atlantic mullet	FT-IR and NIR	- Classification of valuable fish species from cheaper ones<br>- Discrimination between fresh and frozen thawed fillets	- LDA gave a 100% correct classification,<br>- SIMCA a sensitivity higher than 70% and a specificity of 100% were calculated.	[15]
	Cod liver oil (CLO) and vegetable oils (canola, CaO; corn, CO; soybean, SO and walnut, WO)	FT-IR	- Detection of vegetable oils adulterant in cod liver oil	- Classified cod liver oil sample from CaO, CO, SO and WO<br>- Discriminated pure cod liver oil sample from the adulterated sample<br>- r^2 value: 0.99; RMSEC: 0.04-0.82% (v/v) and RMSEP: 1.35-1.75 % (v/v)	[16, 17]
	Five (5) fish oil, six (6) lad, five (5) tallow, five (5) chicken oil, and five (5) suet fat samples	FT-IR	-Classification of different terrestrial animal fats and oils adulteration in fish oil	-PCA analysis showed clear groupings between fish oil, ruminant fat and oils (tallow and suet) and non-ruminant fat and oils (lard and chicken oil)<br>- PLS-DA analysis discriminated adulterated sample (chicken oil, lard, tallow and suet) in fish oil with >95% of the validation accuracy<br>- Detection limits of chicken oil, lard, tallow and suet in fish oil were 0.6%, 0.8%, 2% and 3%, respectively.	[18]
Quality control/ freshness	150 bighead carp	NIRS	- Determination of freshness in various fish samples	- Prediction models (PLSR) of freshness parameter (pH, TBARS, TVB-N, and K) performed well with high (r^2 = 0.945, 0.954, 0.932, 0.807) and low error of prediction (RMSEP = 0.081, 0.107, 2.099, 6.509), respectively	[19]
	100 fish oils	FT-NIR<br>FT-IR	-Identification of quality assessment of fish oil<br>-Determination of lipid oxidation by Artificial Neural Networks (ANN) model	-Combined MIR/NIR data yielded the most reliable ANN model for anisidine (AnV) (r^2= 0.99; RMSEP = 0.74%) detection<br>- MIR data provided acceptable model for acid value (AV) ($r^2$of 0.988 and an RMSEP of 0.43 mg NaOH g^{-1}) detection	[20]
	Rainbow trout fillet (raw and cooked)	NIR	- Investigation of NIR spectroscopy performance for the quality assessment of raw and cooked rainbow trout fillet.	- NIR spectroscopy classified the samples based on their rearing farm<br>- higher classification accuracy obtained using data fusion approach compare to individual model	[21]
	Pacific oysters (*Crassostrea gigas*)	NIR	- Investigation of the freshness	- PLS model revealed acceptable coefficient value (r^2 = 0.8; ratio of SECV to the error range, RER = 5.37) for days of storage and odour (r^2 = 0.77, RER = 7.77)	[22]

				- indicated storage time (sample odor) as (good indicators of freshness than its color	
	Chub mackerel (*Scomber japonicus*)	NIR	- Determination of freshness under different storage temperature	- The samples prepared at 3-Kda ultrafiltrated fraction showed spectral features at 1,379.3 to 1,388.9 nm, involving spectral differences with changes in the storage period.<br>- PLSR model showed greater correlation with coefficient (r^2 = 0.98) for the NIR data and (r^2 = 0.99) for high-performance liquid chromatography (HPLC)data, with cadaverine contents.	[23]
Structural analysis of chemical constituents in marine products	Teleostean fish (chorion /eggshell)	FT-Raman, ATR-IR	- Investigation of the secondary structure features of chorion proteins	- FT-Raman and ATR-IR spectroscopy evaluated the secondary structure of chorion proteins by confirming the abundance of antiparallel β-pleated sheet in eggshell proteins	[24]
	Tilapia (*Oreochromis niloticus*)	FT-IR, Raman	- Investigation of the effect of comminution conditions on the structure of fish protein paste and gels made from surimi and fish protein isolate (FPI)	- Both spectroscopic analysis results found higher degree of unfolding protein structure in FPI and surimi chopped at high temerature (25^0 C) for long time (18 min) compared to low temperature (5^0 C) in a short time (6 min)	[25]
	Lean fish: hake fillets	- FT-IR<br>FT-Raman	- Characterization of lipids class<br>- Investigation of lipids oxidation during frozen storage	-FT-IR data showed significant intensity variation in carbonyl band from 1712.14 ± 0.68 cm^{-1} to 1710.75 ± 0.20 cm^{-1} during frozen storage at -10° C within 5 - 20 weeks, indicating lipid hydrolysis<br>- Lipid oxidation was identrified by Raman analysis at the band shifting of 1658 cm^{-1}, attributed to conjugated dienes development	[26]
	Six different species: sixty-four (64) fish	- Raman microscopy	- Investigation the species discrimination and quality assessment of fish freshness under various freezing/thawing condition	- PCA model enabled to cluster three different batches of samples and determined freshness of the samples<br>- In case of horse mackerel samples, noticeable changes found between fresh and twice-frozen-thawed samples. The reduction of Raman band intensity indicated the alteration of lipid structure.	[27]
	Seabream fillets (5 experimental treatments: 0,1,3,5,7 freeze- thaw cycle)	Raman	-Evaluation of alteration of protein structure, water-protein interaction and physiological properties	-Raman spectral analysis indicated the alteration of secondary protein structure gradually during multiple freeze-thaw cycles	[28]

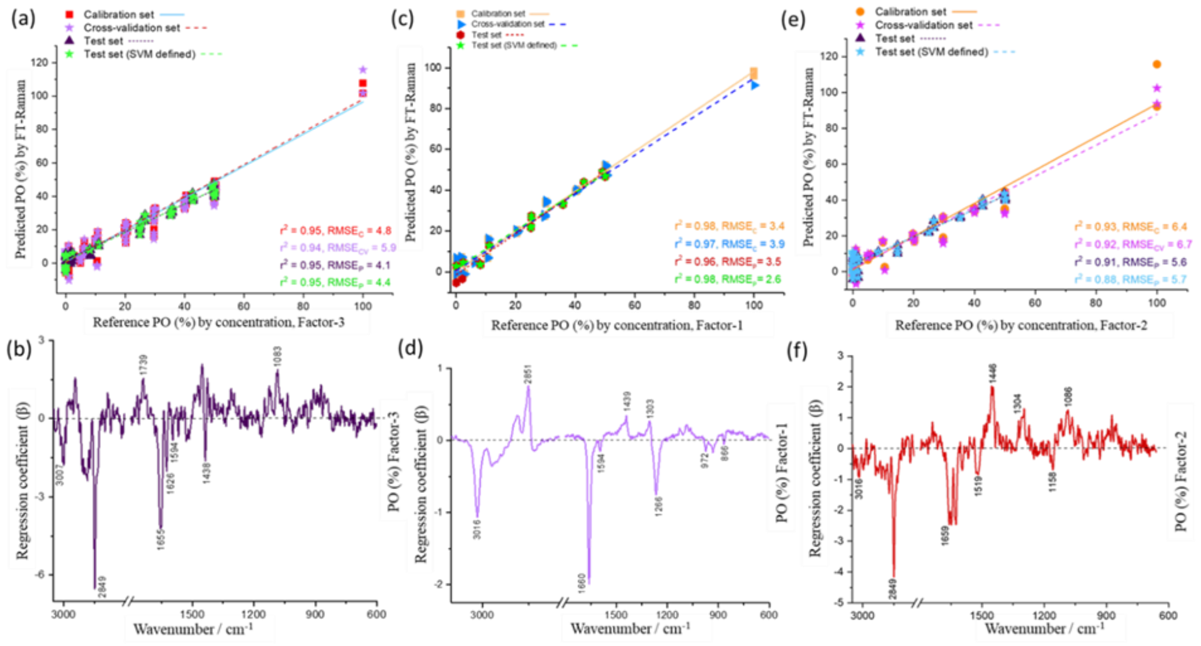

Figure S1. PLSR calibration lines and regression coefficients for quantitative prediction of PO concentration in global (a,b); individual CLO(c,d) and SO (e,f) by Raman.

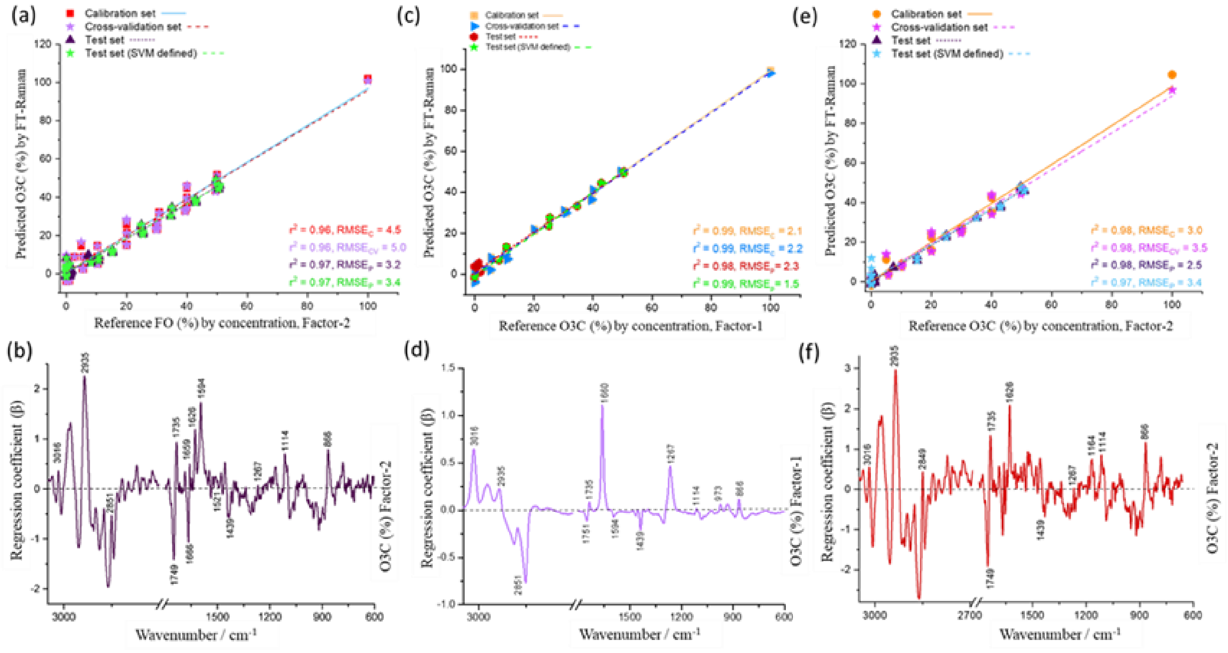

Figure S2. PLSR calibration lines and regression coefficients for quantitative prediction of O3C concentration in global (a,b); individual CLO (c,d) and SO (e,f) by Raman.

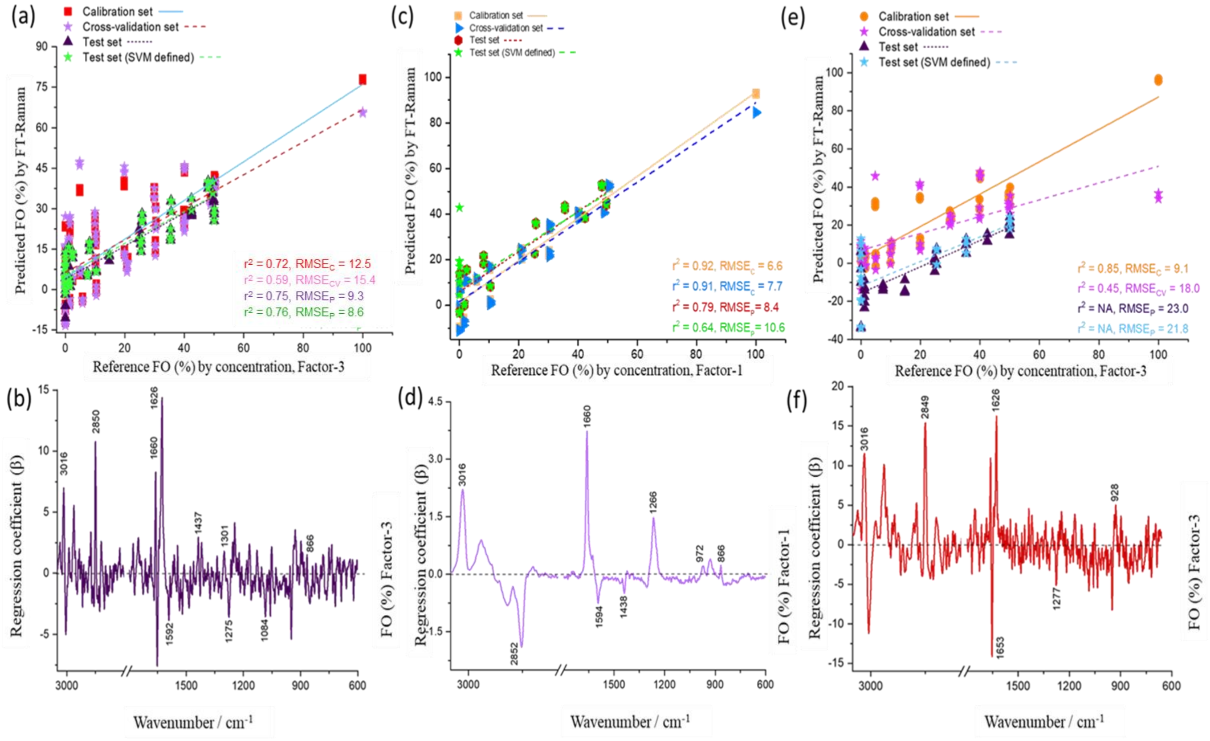

Figure S3. PLSR calibration lines and regression coefficients for quantitative prediction of FO concentration in global (a,b); individual CLO(c,d) and SO (e,f) by Raman.

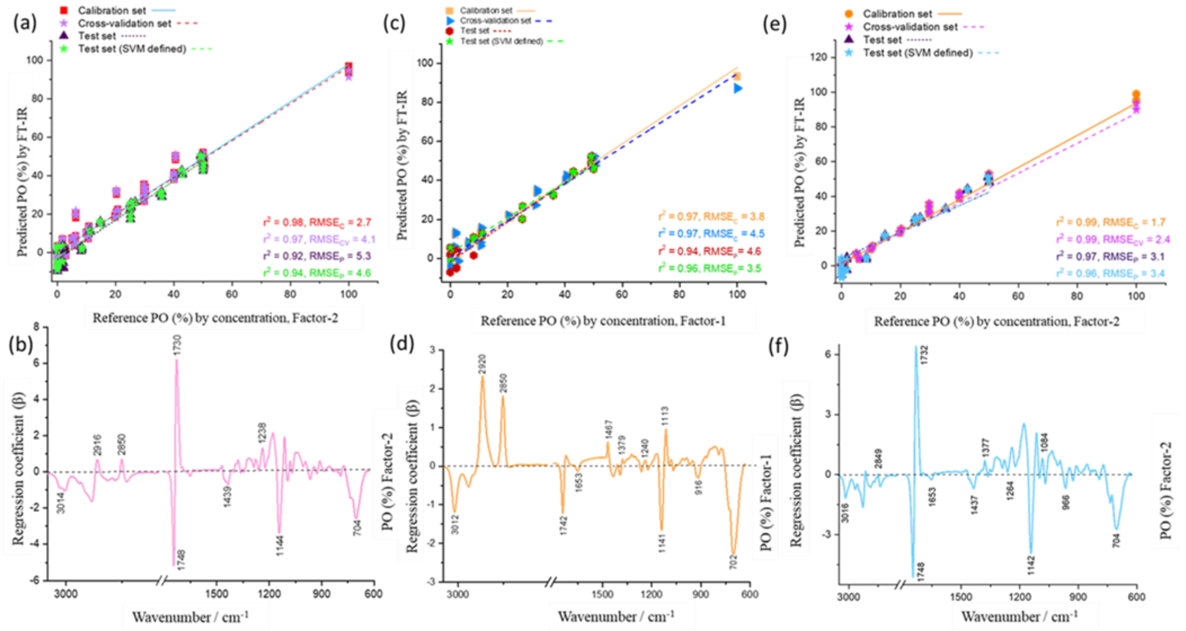

Figure S4. PLSR calibration lines and regression coefficients for quantitative prediction of PO concentration in global (a,b); individual CLO (c,d) and SO (e,f) by IR

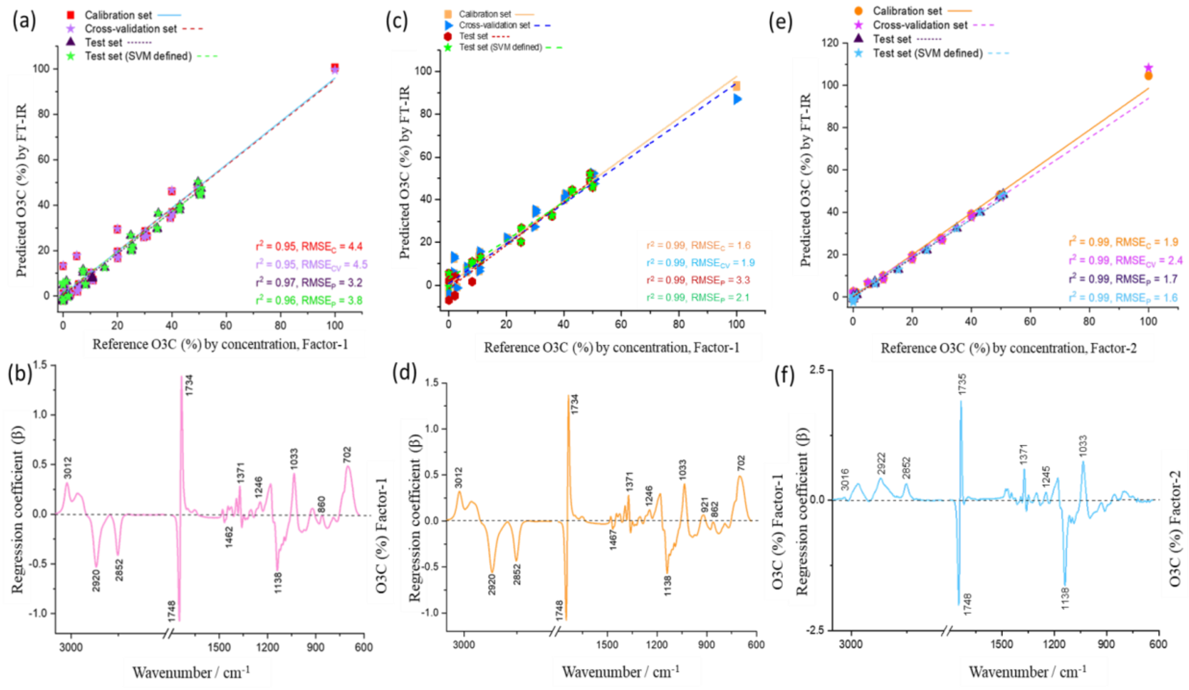

Figure S5. PLSR calibration lines and regression coefficients for quantitative prediction of O3C concentration in global (a,b); individual CLO(c,d) and SO (e,f) by IR.

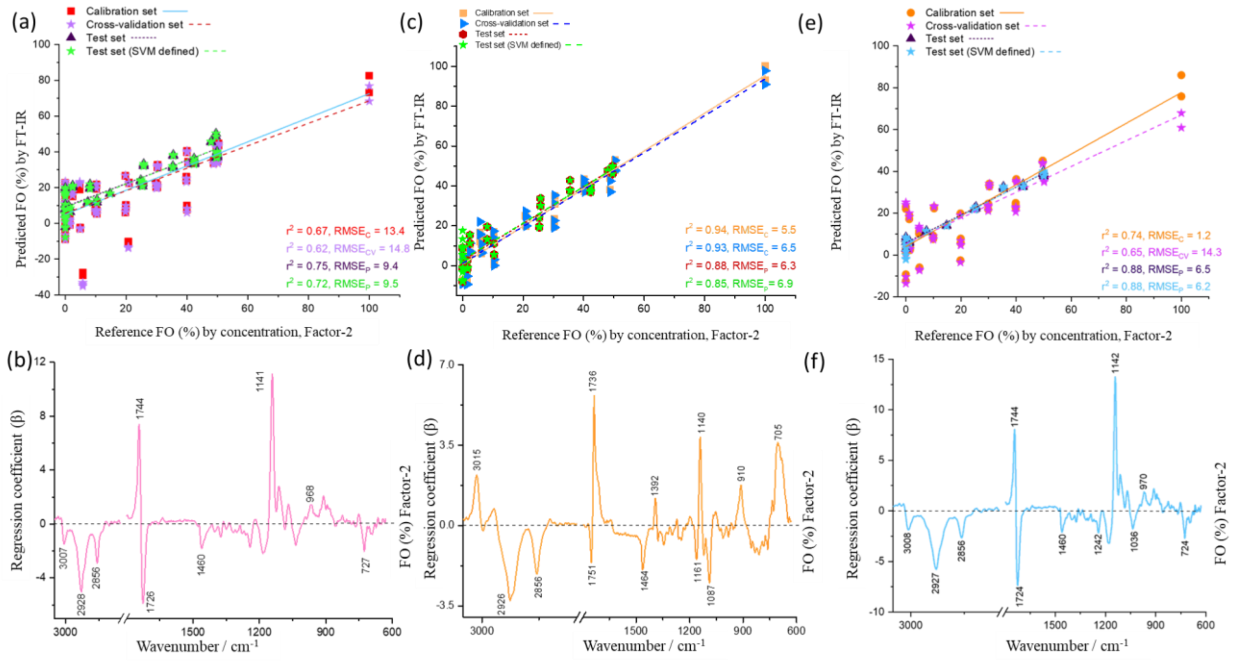

Figure S6. PLSR calibration lines and regression coefficients for quantitative prediction of FO concentration in global (a,b); individual CLO(c,d) and SO (e,f) by IR.

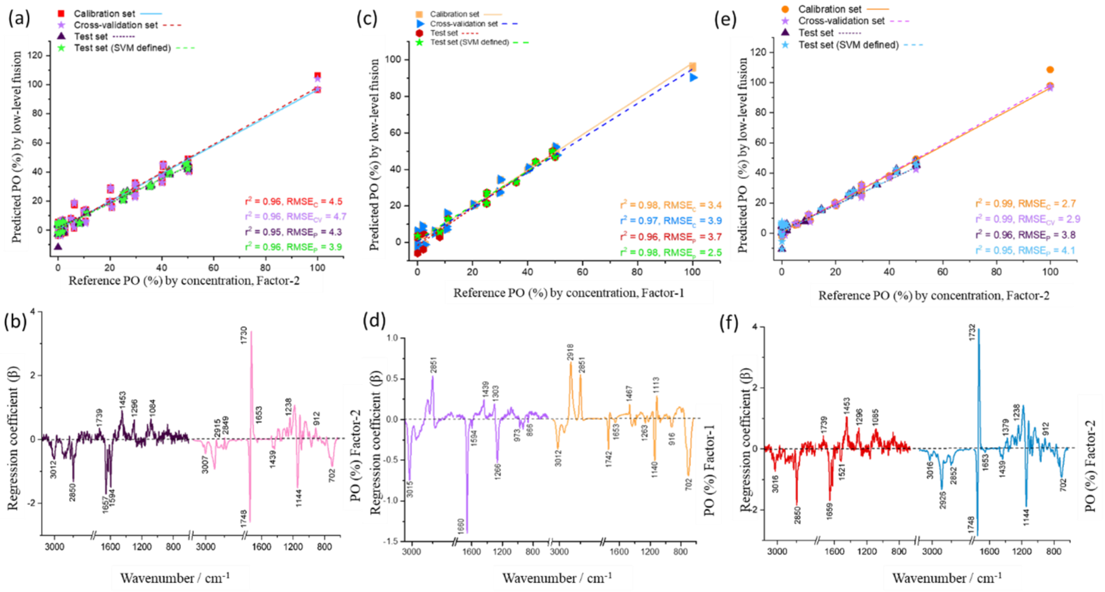

Figure S7. PLSR calibration lines and regression coefficients for quantitative prediction of PO concentration in global (a,b); individual CLO (c,d) and SO (e,f) by Low-level fusion.

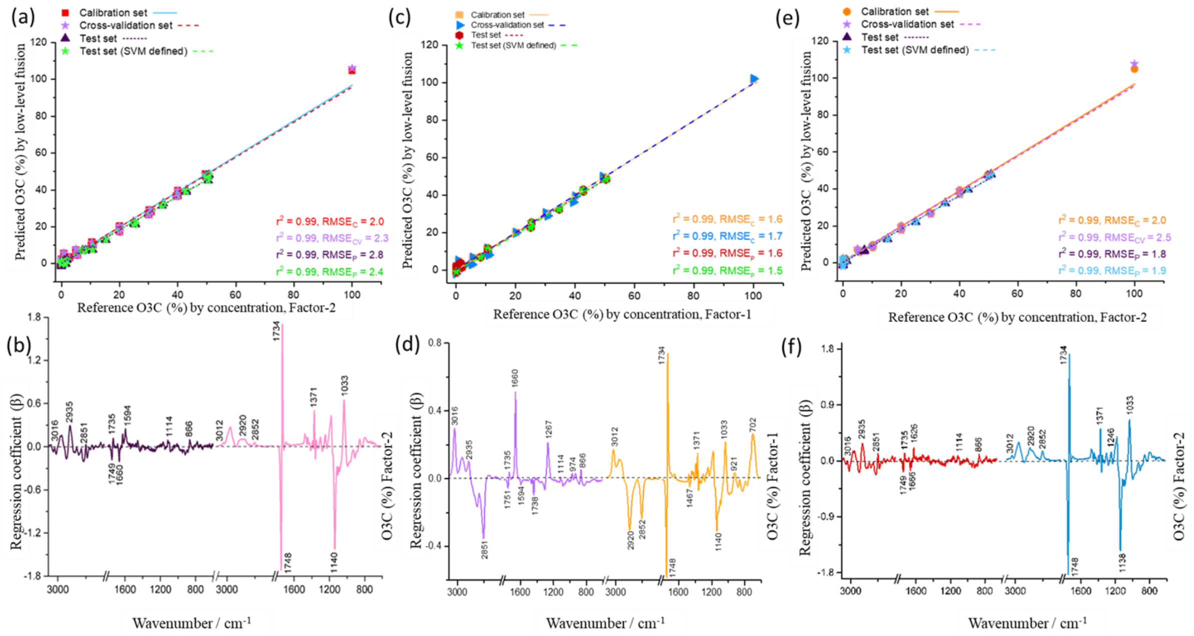

Figure S8. PLSR calibration lines and regression coefficients for quantitative prediction of O3C concentration in global (a,b) ;individual CLO (c,d) and SO (e,f) by Low-level fusion.

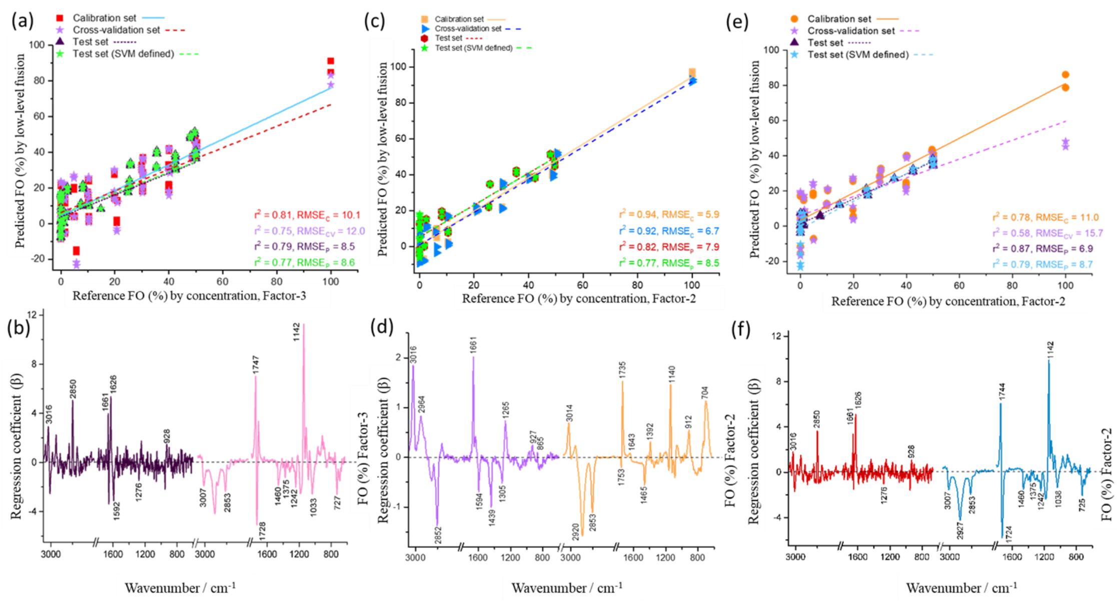

Figure S9. PLSR calibration lines and regression coefficients for quantitative prediction of FO concentration in global (a,b) ;individual CLO (c,d) and SO (e,f) by Low-level fusion.

References

1. Miller, M.R., et al., *Application of a Fourier transform—near infrared reflectance spectroscopy method for the rapid proximate analysis of the greenshell mussel (Perna canaliculus) and king (Chinook) salmon (Oncorhynchus tshawytscha).* Aquaculture Research, 2019. **50**(6): p. 1668-1677.
2. Brown, M.R., et al., *Application of Near-Infrared Reflectance Spectroscopy for the Rapid Chemical Analysis of Sydney Rock Oyster (Saccostrea Glomerata) and Pacific Oyster (Crassostrea gigas).* Journal of Shellfish Research, 2012. **31**(4): p. 1051-1060.
3. Sarabia, G.M. and B. Sharma, *Non-destructive raman spectroscopic determination of freshwater mollusk composition, growth, and damage repair.* Analyst, 2021. **146**(20): p. 6288-6296.
4. Prado, E., et al., *Prediction of fatty acids composition in the rainbow trout Oncorhynchus mykiss by using Raman micro-spectroscopy.* Analytica Chimica Acta, 2021: p. 339212.
5. Marquardt, B.J. and J.P. Wold, *Raman analysis of fish: a potential method for rapid quality screening.* LWT-Food Science and Technology, 2004. **37**(1): p. 1-8.
6. Cascant, M.M., et al., *Determination of fatty acids and lipid classes in salmon oil by near infrared spectroscopy.* Food chemistry, 2018. **239**: p. 865-871.
7. Killeen, D.P., et al., *Raman spectroscopy of fish oil capsules: polyunsaturated fatty acid quantitation plus detection of ethyl esters and oxidation.* Journal of agricultural and food chemistry, 2017. **65**(17): p. 3551-3558.
8. Afseth, N.K., J.P. Wold, and V.H. Segtnan, *The potential of Raman spectroscopy for characterisation of the fatty acid unsaturation of salmon.* Analytica Chimica Acta, 2006. **572**(1): p. 85-92.
9. Bekhit, M.Y., B. Grung, and S.A. Mjøs, *Determination of omega-3 fatty acids in fish oil supplements using vibrational spectroscopy and chemometric methods.* Applied spectroscopy, 2014. **68**(10): p. 1190-1200.
10. Putri, A.R., A. Rohman, and S. RIYANTO, *Authentication of patin (Pangasius micronemus) fish oil adulterated with palm oil using FTIR spectroscopy combined with chemometrics.* International Journal of Applied Pharmaceutics, 2019: p. 195-199.
11. Amorim, T.L., et al., *ATR-FTIR and Raman Spectroscopies Associated with Chemometrics for Lipid Form Evaluation of Fish Oil Supplements: A Comparative Study.* ACS Food Science & Technology, 2021. **1**(3): p. 318-325.
12. Boughattas, F., B. Le Fur, and R. Karoui, *Mid infrared spectroscopy coupled with chemometric tools for qualitative analysis of canned tuna with sunflower medium.* Journal of Food Composition and Analysis, 2020. **91**: p. 103519.
13. Lv, H., et al., *Classification of freshwater fish species by linear discriminant analysis based on near infrared reflectance spectroscopy.* Journal of Near Infrared Spectroscopy, 2017. **25**(1): p. 54-62.
14. Dominguez-Vidal, A., et al., *Authentication of canned fish packing oils by means of Fourier transform infrared spectroscopy.* Food Chemistry, 2016. **190**: p. 122-127.
15. Alamprese, C. and E. Casiraghi, *Application of FT-NIR and FT-IR spectroscopy to fish fillet authentication.* LWT-Food Science and Technology, 2015. **63**(1): p. 720-725.
16. Rohman, A. and Y.B. Che Man, *Analysis of cod-liver oil adulteration using Fourier transform infrared (FTIR) spectroscopy.* Journal of the American Oil Chemists' Society, 2009. **86**(12): p. 1149.
17. Rohman, A. and Y.B. Che Man, *Application of Fourier transform infrared (FT-IR) spectroscopy combined with chemometrics for authentication of cod-liver oil.* Vibrational Spectroscopy, 2011. **55**(2): p. 141-145.
18. Lingzhi, X., et al., *Discriminant analysis of terrestrial animal fat and oil adulteration in fish oil by infrared spectroscopy.* International Journal of Agricultural and Biological Engineering, 2016. **9**(3): p. 179-185.
19. Zhou, J., et al., *Evaluation of freshness in freshwater fish based on near infrared reflectance spectroscopy and chemometrics.* Lwt, 2019. **106**: p. 145-150.
20. Giese, E., et al., *Determining quality parameters of fish oils by means of 1H nuclear magnetic resonance, mid-infrared, and near-infrared spectroscopy in combination with multivariate statistics.* Food Research International, 2018. **106**: p. 116-128.
21. Dalle Zotte, A., et al., *Authentication of raw and cooked freeze-dried rainbow trout (Oncorhynchus mykiss) by means of near infrared spectroscopy and data fusion.* Food Research International, 2014. **60**: p. 180-188.
22. Madigan, T., et al., *The use of rapid instrumental methods to assess freshness of half shell Pacific oysters, Crassostrea gigas: A feasibility study.* Innovative Food Science & Emerging Technologies, 2013. **19**: p. 204-209.
23. Shim, K. and Y. Jeong, *Freshness Evaluation in Chub Mackerel (Scomber japonicus) Using Near-Infrared Spectroscopy Determination of the Cadaverine Content.* Journal of Food Protection, 2019. **82**(5): p. 768-774.
24. Iconomidou, V.A., et al., *Secondary structure of chorion proteins of the teleostean fish Dentex dentex by ATR FT-IR and FT-Raman spectroscopy.* Journal of structural biology, 2000. **132**(2): p. 112-22.
25. Kobayashi, Y., S.G. Mayer, and J.W. Park, *FT-IR and Raman spectroscopies determine structural changes of tilapia fish protein isolate and surimi under different comminution conditions.* Food Chemistry, 2017. **226**: p. 156-164.
26. Sanchez-Alonso, I., P. Carmona, and M. Careche, *Vibrational spectroscopic analysis of hake (Merluccius merluccius L.) lipids during frozen storage.* Food Chemistry, 2012. **132**(1): p. 160-167.
27. Velioglu, H.M., H.T. Temiz, and I.H. Boyaci, *Differentiation of fresh and frozen-thawed fish samples using Raman spectroscopy coupled with chemometric analysis.* Food Chemistry, 2015. **172**: p. 283-90.
28. Cao, M., et al., *The impact of recrystallisation on the freeze-thaw cycles of red seabream (Pagrus major) fillets.* International Journal of Food Science & Technology, 2018. **54**(5): p. 1642-1650.
